# Real‐Time Monitoring of Multitarget Antimicrobial Mechanisms of Peptoids Using Label‐Free Imaging with Optical Diffraction Tomography

**DOI:** 10.1002/advs.202302483

**Published:** 2023-06-21

**Authors:** Minsang Kim, Yeongmi Cheon, Dongmin Shin, Jieun Choi, Josefine Eilsø Nielsen, Myeong Seon Jeong, Ho Yeon Nam, Sung‐Hak Kim, Reidar Lund, Håvard Jenssen, Annelise E. Barron, Seongsoo Lee, Jiwon Seo

**Affiliations:** ^1^ Department of Chemistry Gwangju Institute of Science and Technology (GIST) 123, Cheomdangwagi‐ro, Buk‐gu Gwangju 61005 Republic of Korea; ^2^ Gwangju Center Korea Basic Science Institute (KBSI) 49, Dosicheomdansaneop‐ro, Nam‐gu Gwangju 61751 Republic of Korea; ^3^ Laboratory of Molecular Biochemistry Chonnam National University 77, Yongbong‐ro, Buk‐gu Gwangju 61186 Republic of Korea; ^4^ Department of Microbiology and Molecular Biology Chungnam National University 99, Daehak‐ro, Yuseong‐gu Daejeon 34134 Republic of Korea; ^5^ Department of Science and Environment Roskilde University Universitetsvej 1 Roskilde 4000 Denmark; ^6^ Department of Bioengineering, Schools of Medicine and Engineering Stanford University 443 Via Ortega Stanford California 94305 United States; ^7^ Chuncheon Center Korea Basic Science Institute (KBSI) 1, Kangwondaehak‐gil, Chuncheon‐si Gangwon‐do 24341 Republic of Korea; ^8^ Department of Chemistry University of Oslo Problemveien 7 Oslo 0315 Norway; ^9^ Department of Systems Biotechnology Chung‐Ang University Anseong‐si Gyeonggi‐do 17546 Republic of Korea

**Keywords:** antimicrobial peptide, antimicrobial peptoid, multitarget mechanism, optical diffraction tomography

## Abstract

Antimicrobial peptides (AMPs) are promising therapeutics in the fight against multidrug‐resistant bacteria. As a mimic of AMPs, peptoids with *N*‐substituted glycine backbone have been utilized for antimicrobials with resistance against proteolytic degradation. Antimicrobial peptoids are known to kill bacteria by membrane disruption; however, the nonspecific aggregation of intracellular contents is also suggested as an important bactericidal mechanism. Here,structure‐activity relationship (SAR) of a library of indole side chain‐containing peptoids resulting in peptoid **29** as a hit compound is investigated. Then, quantitative morphological analyses of live bacteria treated with AMPs and peptoid **29** in a label‐free manner using optical diffraction tomography (ODT) are performed. It is unambiguously demonstrated that both membrane disruption and intracellular biomass flocculation are primary mechanisms of bacterial killing by monitoring real‐time morphological changes of bacteria. These multitarget mechanisms and rapid action can be a merit for the discovery of a resistance‐breaking novel antibiotic drug.

## Graphical abstract

Quantitative morphological analyses of live bacteria treated with melittin, buforin‐II, and hit peptoid (**29**) in a label‐free manner using ODT) are performed. With antimicrobial peptoid **29**, it is unambiguously demonstrated that both membrane disruption and intracellular biomass flocculation are primary mechanisms of bacterial killing by real‐time ODT imaging.

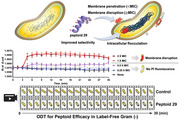

## 1 Introduction

Conventional antibiotics gradually lose their efficacy due to the emergence of multi‐drug resistant (MDR) bacteria.^[^ ^1^ ^]^ The steady decline in the introduction of new antibiotic compounds into clinical use leaves the human population vulnerable to MDR pathogens.^[^ ^2^ ^]^ Given the increasing threat posed by MDR bacteria, antimicrobial agents simultaneously engaging multiple targets have been highlighted as a potential strategy for overcoming resistance.^[^ ^3^ ^]^ AMPs have protected living organisms with their ability to kill invading pathogens.^[^ ^4^ ^]^ AMPs represent an innate immune defense mechanism that evolved to eliminate a broad‐spectrum of potential pathogens, typically affecting multiple targets within the invading organism.^[^ ^3^, ^5^ ^]^

Natural AMPs commonly have cationic and amphipathic properties, facilitating their interaction with bacterial membrane components such as lipopolysaccharides (LPS), lipoteichoic acids (LTA), and anionic phospholipids.^[^ ^6^ ^]^ Upon interaction, some AMPs disrupt or depolarize the bacterial membrane during the drug action. Some AMPs with intrinsic cell‐penetrating properties inhibit intracellular functions by targeting nucleotides (DNA and RNA) and proteins, or by causing aggregation of these intracellular biomass.^[^ ^5^, ^7^ ^]^

Antibiotic drug candidates have been developed by engineering natural AMPs; for example, omiganan and TC19 are derivatives of indolicidin, a linear tryptophan‐rich AMP.^[^ ^8^ ^]^ Although AMPs represent a promising molecular platform for antibiotic drug discovery, they have notable shortcomings. The most important of these is the poor in vivo stability and potential immunogenicity of the compounds, limiting their applications in systemic circulations.^[^ ^9^ ^]^ For example, omiganan was limited to topical administrations in late phase clinical trials. As an alternative approach, mimicry of AMPs using peptidomimetic scaffolds that overcome these issues has emerged, and peptoids are highlighted in this context. Peptoids are heterooligomers based on an *N*‐substituted glycine backbone.^[^ ^10^ ^]^ Peptoids are less prone to in vivo enzymatic degradation^[^ ^11^ ^]^ and are generally more membrane permeable than natural peptides.^[^ ^11^, ^12^ ^]^ The helical and amphipathic features of natural magainin have been adopted in peptoids, leading to the discovery of peptoid **1** with potent activity against a broad‐spectrum of pathogens (**Table** **1**).^[^ ^7^, ^13^ ^]^ Following peptoid **1**, various antimicrobial peptoids have been reported employing distinct design strategies aiming to improve antimicrobial activity and selectivity. These approaches included macrocyclization,^[^ ^14^ ^]^ hydrocarbon tail conjugation,^[^ ^15^ ^]^ helicity modulation,^[^ ^16^ ^]^ and the incorporation of novel side chains incorporation such as indoles or aryl halides.^[^ ^15^, ^17^ ^]^

**Table 1:** Sequences and properties of *N*Trp‐containing antimicrobial peptoids

#	Sequence	HPLC elution (%ACN)	Net charge
**1**	H‐***N*Lys**‐*N*spe‐*N*spe‐***N*Lys**‐*N*spe‐*N*spe‐***N*Lys**‐*N*spe‐*N*spe‐***N*Lys**‐*N*spe‐*N*spe‐NH_2_	54.5	+4
**10**	H‐***N*Lys**‐*N*spe‐*N*Trp‐***N*Lys**‐*N*spe‐*N*spe‐***N*Lys**‐*N*spe‐*N*spe‐***N*Lys**‐*N*spe‐*N*spe‐NH_2_	54.9	+4
**11**	H‐***N*Lys**‐*N*spe‐*N*spe‐***N*Lys**‐*N*spe‐*N*spe‐***N*Lys**‐*N*spe‐*N*spe‐***N*Lys**‐*N*Trp‐*N*Trp‐NH_2_	54.0	+4
**14**	H‐***N*Lys**‐*N*Trp‐*N*Trp‐***N*Lys**‐*N*spe‐*N*spe‐***N*Lys**‐*N*spe‐*N*spe‐***N*Lys**‐*N*spe‐*N*spe‐NH_2_	55.6	+4
**29**	H‐***N*Lys**‐*N*spe‐*N*spe‐***N*Lys**‐*N*spe‐*N*spe‐***N*Lys**‐*N*Lys‐*N*spe‐***N*Lys**‐*N*Trp‐*N*Trp‐NH_2_	48.6	+5
**32**	H‐***N*Lys**‐*N*Trp‐*N*Trp‐***N*Lys**‐*N*spe‐*N*spe‐***N*Lys**‐*N*spe‐*N*spe‐***N*Lys**‐*N*Lys‐*N*spe‐NH_2_	48.4	+5
**45**	H‐***N*Lys**‐*N*spe‐*N*spe‐***N*Lys**‐*N*spe‐*N*spe‐***N*Lys**‐*N*Lys‐*N*spe‐***N*Lys**‐*N*pm‐*N*pm‐NH_2_	53.1	+5
Melittin	H‐GIGAVLKVLTTGLPALISWIKRKRQQ‐NH_2_	55.4	+5
Buforin‐II	H‐TRSSRAGLQFPVGRVHRLLRK‐NH_2_	37.6	+6
Omiganan	H‐ILRWPWWPWRRK‐NH_2_	45.7	+4
Pexiganan	H‐GLGKFLKKAKKFGKAFVKILKK‐NH_2_	43.5	+9

To study the mechanisms of antimicrobial peptides/peptoids, phenotypic changes of bacteria have been investigated utilizing fluorescence confocal microscopy,^[^ ^16^, ^18^ ^]^ phase contrast microscopy,^[^ ^19^ ^]^ electron microscopy (EM),^[^ ^7b^ ^]^ and atomic force microscopy (AFM).^[^ ^7^, ^16^, ^20^ ^]^ However, the use of fluorescent imaging for the long‐term monitoring of live cells is limited by photobleaching of dyes and their photocytotoxicity.^[^ ^21^ ^]^ Conventional EM is not suitable for live cell imaging because of the required fixation and sectioning steps, and is limited to the creation of two‐dimensional (2D) images. Cryo‐EM can provide tomographic images; however, it is technically challenging for nonexperts. AFM is capable of time‐lapse monitoring in a label‐free manner, but it is limited by the need to obtain tomographic images and the difficulty of preparing immobilized microbial samples.^[^ ^22^ ^]^

ODT has been developed as an advanced quantitative phase imaging (QPI) technique for the long‐term real‐time monitoring of living cells. ODT allows noninvasive imaging without any pretreatment steps or additional labeling, facilitating the real‐time monitoring of phenotypic changes over longer periods. Moreover, ODT enables reconstructed images of biological samples based on three‐dimensional (3D) refractive index (RI) maps that attribute inherent values to each intracellular component, and can characterize morphological and biophysical properties at submicrometer resolution.^[^ ^23^ ^]^ The ability to generate 3D RI distribution maps and conduct time‐lapse monitoring are critical features for the analysis of morphological changes in cellular volume and dry mass at the level of individual cells. As a real‐time and label‐free imaging technique, 3D ODT has been utilized in immunology,^[^ ^24^ ^]^ cell biology,^[^ ^23^, ^25^ ^]^ and drug discovery.^[^ ^26^ ^]^ Morphological changes affecting bacterial cells due to external stressors have also been monitored by 3D ODT.^[^ ^23b,d^ ^]^ However, the investigation of antimicrobial agents and their mechanisms of action using ODT remains a mostly unexplored field.

The work described here demonstrates that indole side chain‐containing antimicrobial peptoids affect multiple target pathways simultaneously. Using real‐time label‐free 3D ODT imaging, we first assessed the suitability of this approach for the investigation of antimicrobial mechanisms. To do so, we first selected two well‐known natural peptides for analysis via ODT. Melittin is a widely studied membrane disrupting peptide utilized to develop synthetic lytic AMPs while buforin‐II is a representative penetrating AMP. By conducting these preliminary studies, we first developed a quantitative method for the morphological analysis of bacterial cells. Next, we investigated the mechanism of action of the potent and selective peptoid **29**, chosen from the screening of an indole side chain‐containing peptoid library of 66 compounds. Finally, the specific RI values of gram‐negative bacteria treated with peptoid **29** were analyzed through time‐lapse monitoring using 3D ODT. In combination with fluorescence and EM imaging data, bacterial ODT analysis demonstrated the flocculation of intracellular biomass (e.g., proteins and nucleic acids) as well as membrane disruption upon exposure of the cells to peptoid **29**. These results confirm the multitarget mechanism of action of peptoid **29**. The ability of ODT to provide label‐free real‐time monitoring of live cells indicates that it is an eminently useful tool for screening antibiotic compounds that cause morphological and biophysical changes in the membrane and/or the intracellular components of bacteria. In these situations, ODT can establish the mechanism(s) underlying the antimicrobial action of the tested compounds.

## 2 Results and Discussion

### 2.1 Morphological Analysis of *E. coli* Treated with AMPs Using 3D ODT and EM

Although the utility of 3D ODT imaging technique has been demonstrated in diverse areas of cell biology, those studies almost exclusively involved eukaryotic cells. For the much smaller bacterial cells, preliminary observations on the morphological changes after beta‐lactam antibiotic treatment were only reported recently.^[^ ^23b^ ^]^ However, in‐depth antibacterial mechanism studies using 3D ODT have not been previously attempted. To make such studies possible, the optimal experimental conditions for the measurement of RI distributions in bacterial cells needed to be established and the interpretation of imaging data needed validation. First, we selected two representative AMPs, melittin, and buforin‐II, with well‐established mechanisms of action data, and conducted preliminary experiments to determine whether 3D ODT was a valid technique for the monitoring of bacterial cell death. Melittin is a membrane disrupting peptide,^[^ ^6b^ ^]^ while buforin‐II is membrane permeable and inhibits cellular function by targeting intracellular components, including DNA and RNA.^[^ ^7a^ ^]^ After treatment with these AMPs, we monitored the morphology of *E. coli* to detect changes in RI distribution. In addition, Scanning electron microscopy (SEM) and transmission electron microscopy (TEM) images were obtained to identify the mode of action by comparisons with previously reported EM data.^[^ ^7^, ^27^ ^]^

**Figure 1:**
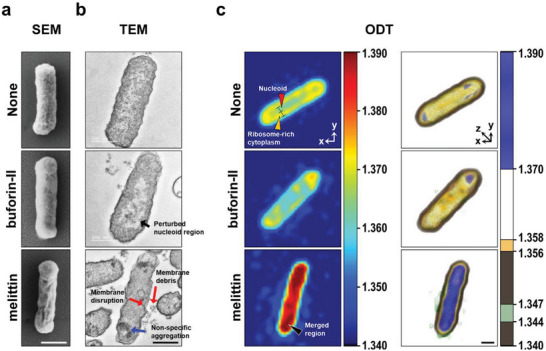
Microscopic imaging of *E. coli* ATCC 25922 treated with buforin‐II or melittin (25 µM, 2 × MIC). a) Scanning electron micrographs. b) Transmission electron micrographs. Black arrow: perturbed nucleoid region, red arrow: membrane disruption and debris, and blue arrow: nonspecific aggregation. c) RI‐based ODT imaging. Data were captured at 30 min after AMP injection. 2D projection (left) and 3D rendered images (right). * means RI = ≈1.360 region suggested as a nucleoid region. The color maps denote several colors depending on RI values. Scale bars = 500 nm. Red arrowhead: nucleoid, yellow arrowhead: cytoplasm with ribosomes, and black arrowhead: merged region.

As shown in **Figure** **1a,b**, SEM and TEM images of nontreated *E. coli* showed intact membrane and a uniform nucleoid and cytoplasm. The surface morphology of *E. coli* treated with buforin‐II was similar to that of the nontreated bacteria. However, the irregular internal morphology of the nucleoid region was clearly visible in the TEM image.^[^ ^27a^ ^]^ We assumed that the intact surface morphology and perturbed intracellular image pattern correlated with the mechanism of action of buforin‐II, the disruption of nucleic acid content after the penetration of the bacterial membrane. When *E. coli* were treated with melittin, EM images depicted damaged surface morphology due to membrane disruption. A nonspecific aggregation in the bacterial cytoplasm was also observed in comparison to nontreated *E. coli*.^[^ ^7^, ^27^ ^]^ However, these conventional TEM images are limited in that (1) real‐time live cell imaging is not possible and (2) the intracellular components of chemically fixed cells can often appear as an irregular structure. Therefore, we obtained 2D cross‐sectional images of live bacterial cells using ODT (Figure 1c) and compared these with the EM images.

The morphology of *E. coli* was represented by RI values ranging from 1.340 to 1.390. It was previously reported that in intact bacteria the nucleoid was elliptical in shape and was radially confined to the center of the cell, and that ribosomes could be identified around the nucleoid, with the two structures clearly separated in the cytoplasm.^[^ ^19^, ^28^ ^]^ 2D cross‐sectional images of nontreated bacteria (Figure 1c, top panel) showed this spatial segregation of the nucleoid and ribosomes. The center of the cell, indicated with the azure color and the red arrowhead (RI = ≈1.360), was assumed to be the nucleoid. The more peripheral ribosomes and proteins were depicted by the yellow color (RI = ≈1.370). After treatment with buforin‐II, the boundary of the nucleoid (azure) was disrupted and overall RI values were decreased. In the case of melittin‐treated *E. coli*, the visual distinction between nucleoid and protein‐rich region became blurred and the overall RI values of the intracellular region increased to 1.380–1.390, as depicted by the red/crimson color in the image (black arrowhead).

Next, we obtained 3D reconstructed images based on specific RI values with defined colors in Figure 1c (panels on the right) to monitor changes in the cytoplasm and cell membrane. The RI values were chosen to highlight morphological changes caused by melittin. The merged dense regions inside the cell (shown in blue, RI = 1.370–1.390) and the leakage of cytoplasmic content (shown in green, RI = 1.344–1.347), caused by the disruption of the cell membrane, suggested distinct features when bacteria were treated with a membrane disrupting AMP.^[^ ^7^, ^27^, ^29^ ^]^ With the same RI values (RI = 1.370–1.390 and 1.344–1.347), the 3D reconstructed images of control (nontreated) and buforin‐II treated cells did not show any noticeable differences. Together, these results showed that 3D ODT with RI mapping is an effective analytical tool for the monitoring of bacterial cells without the need for fluorescent labels or any chemical pretreatment before imaging. Thus, this imaging modality appeared to be an ideal tool to clarify the mechanism(s) of action of antimicrobial AMPs targeting the cell membrane or intracellular components of bacteria.

**Figure 2:**
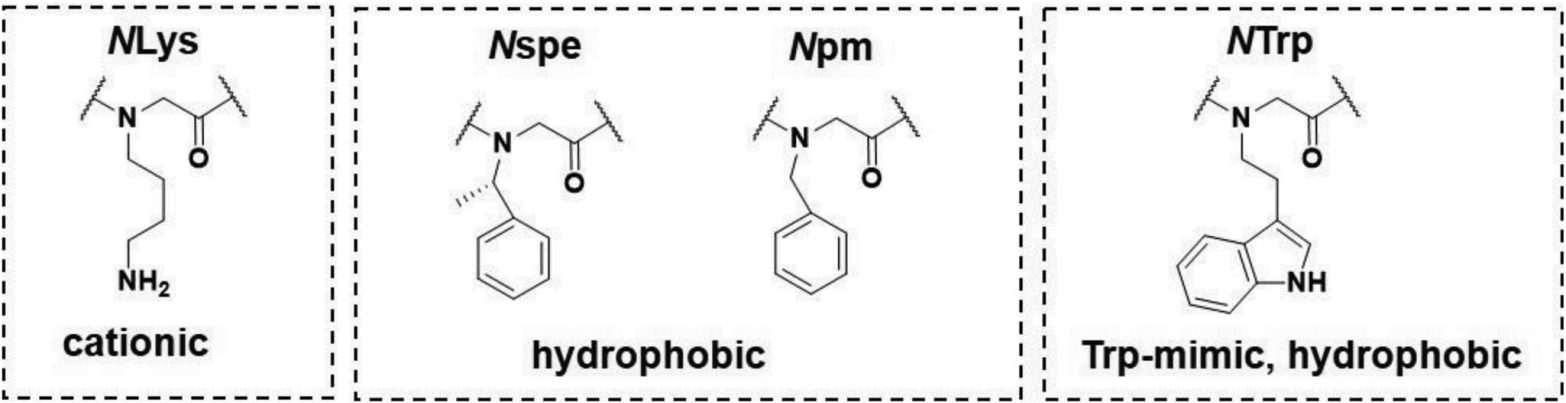
Structures of peptoid monomers and their abbreviations.

### 2.2 Biological Activity Screening and SAR Analysis of *N*Trp‐Containing Peptoid Library

Trp‐rich AMPs exhibit an intrinsic preference for membrane interaction due to the physicochemical properties of the indole ring.^[^ ^30^ ^]^ The hydrophobicity, dipole moment, and hydrogen‐bonding potential of the indole ring lead to a favorable side‐chain alignment at the lipid‐water interface of the membrane, causing the subsequent disruption of ordered phospholipids.^[^ ^31^ ^]^ Recent small‐angle X‐ray scattering (SAXS) studies demonstrated that the preferential positioning of indolicidin at the membrane interface was promoted by indole‐lipid head group interactions significantly accelerating lipid dynamics.^[^ ^32^ ^]^ Especially, it was reported that the WW motif in natural or synthetic AMPs was associated with membrane insertion, perturbation, and permeability that correlated directly with antimicrobial activity.^[^ ^33^ ^]^

In an attempt to identify antimicrobial peptoids with improved potency and selectivity, a library of 66 peptoids, including 49 novel, indole side chain (*N*Trp)‐containing peptoids and 17 control peptoids, was synthesized (Table S1, Supporting Information). The antimicrobial activity against gram‐negative *E. coli* and gram‐positive *S. aureus* was evaluated by determining their minimal inhibitory concentrations (MIC). The selectivity of these compounds for bacterial cells was evaluated by assessing their hemolytic activity (Table S2, Supporting Information). After a SAR analysis, described in the supporting information (Section 5), 7 peptoids were selected from the library for further testing. The features of these, together with relevant control AMPs, are shown in **Table** **1** and **Figure** 2. The previously reported peptoid 1 was used as a positive control.^[^ ^13^ ^]^ Peptoid 10 contains one *N*Trp by substituting *N*spe of peptoid **1**, while peptoids **11** and **14** contain two adjacent *N*Trp residues mimicking the WW motif in Trp‐rich AMPs. Although exhibiting highly potent antimicrobial activity, peptoids **10**, **11**, and **14** also lysed erythrocytes even at low concentrations. This was probably due to their hydrophobicity as indicated by their HPLC elution profiles (Tables 1 and 2). Generally, the cytotoxicity of AMPs and antimicrobial peptoids increases with increasing overall hydrophobicity. Therefore, increasing the cationic charge by the incorporation of additional *N*Lys is expected to improve selectivity towards bacterial membranes. Given these considerations, *N*spe to *N*Lys substitutions were made to peptoids **11** and **14**, generating peptoids **28**–**34** (Table S1, Supporting Information). These seven peptoids have the same charge‐to‐length ratio (CTLR of 0.42) and share the common WW motif. Of these compounds, peptoids **29** and **32** retained the antimicrobial activity of the “parent” compounds, while showing significantly improved selectivity (i.e., selectivity index of >15.9 for **29** and 12.3 for **32**). In addition, **29** and **32** showed higher LC_50_ values against both normal human lung fibroblasts (MRC‐5) and human epithelial keratinocyte (HaCaT) than peptoid **1**. In peptoid **45**, the two indole rings of **29** were replaced by phenyl rings, substituting *N*Trp with *N*pm. Although these changes increased hydrophobicity, they resulted in the loss of antimicrobial and hemolytic activities, demonstrating the importance of the WW motif in peptoids **29** and **32**. Additional SAR studies were performed after counter‐ion exchanging the *N*Trp‐containing peptoids (Table S3, Supporting Information). Although antimicrobial activity was maintained following the counter‐ion exchange from TFA to HCl or to acetic acid (AcOH), the impact of this alteration on hemolytic activity and cytotoxicity against eukaryotic cells varied. Notably, changing **29**‐TFA to **29**‐AcOH reduced hemolysis caused by this compound while in vitro antimicrobial activity was maintained.

**Table 2:** Antimicrobial, hemolytic, and cytotoxic activities of peptoids

#	MIC^a)^ (µM)		HC_10_/HC_50_ ^b)^ (µM)	*H*_max_ ^c)^ (100 µM)	Selectivity index*^d)^*	LC_50_ *^e)^* (µM)	
	*E. coli* ATCC25922	*S. aureus* ATCC25923				MRC‐5	HaCaT
**1**	6.3	1.6	8.3/22.9	112.3 ± 0.7	1.3	8.1	7.3
**10**	3.1	1.6	6.6/17.0	108.1 ± 1.7	2.1	nd^f^	nd^f^
**11**	6.3	1.6	8.3/22.2	103.4 ± 3.2	1.3	6.9	nd^f^
**14**	3.1	<0.8	23.6/43.4	90.0 ± 7.3	7.6	9.7	nd^f^
**29**	6.3	6.3	>100/>100	9.8 ± 1.8	>15.9	11.8	18.8
**32**	6.3	3.1	77.7/>100	15.9 ± 1.5	12.3	14.5	17.1
**45**	>25	25	>100/>100	4.5 ± 0.2	nd^f^	nd^f^	nd^f^
Melittin	12.5	≤ 1.6	<3.1/4.0	105.7 ± 17.6	<0.2	nd^f^	nd^f^
Buforin‐II	12.5	12.5	nd^f^	nd^f^	nd^f^	nd^f^	nd^f^
Omiganan	12.5	12.5	>100/>100	0	>8.0	nd^f^	nd^f^
Pexiganan	3.1	≤ 1.6	>100/>100	8.2 ± 0.6	>32.3	nd^f^	nd^f^

^a)^ These concentrations represent mean values of triplicates.

^b)^ HC_10_ and HC_50_ are the concentrations of compounds causing 10% and 50% hemolysis in rat erythrocytes, respectively. These concentrations represent mean values of triplicates.

^c)^ *H*_max_ is the percentage (%) of hemolysis at the highest concentration tested (100 µM).

^d)^ The selectivity index was calculated by dividing HC_10_ by the MIC value against *E. coli* ATCC 25 922.

^e)^ LC_50_ is the concentration of the compounds resulting in 50% of the cells being killed.

^f)^ Not determined.

### 2.3 Antimicrobial Activity Against Pathogenic and MDR Strains

**Table** **3** summarizes the antimicrobial activity of selected peptoids against gram‐positive and gram‐negative MDR bacterial strains. As reported previously, peptoid **1** showed broad‐spectrum antimicrobial activity including activity against MDR strains.^[^ ^34^ ^]^ Peptoid **11** demonstrated similarly potent broad‐spectrum antimicrobial activity. These two peptoids have the same cationic charge and similar hydrophobicity, and their biological activities, including the tendency to cause hemolysis, were also similar. Compared to **11**, peptoid **29** showed less potent antimicrobial activity against gram‐positive strains, but still its MIC values were retained at submicromolar levels. Against gram‐negative bacteria, peptoid **29** exhibited increased activity against *S. typhimurium*, comparable activity against *P. aeruginosa*, and decreased activity against the multi‐drug resistant *E. coli* (ESBL 63 103) and *K. pneumonia* (ESBL 61 962) strains. Compared to peptoid **11**, **29** has an increased cationic charge and is less hydrophobic. These differences led to significantly reduced toxicity against eukaryotic cells (Table 2). Thus, the reduced antimicrobial activity against the two ESBL stains was offset by increased selectivity.

**Table 3:** Antimicrobial activity of selected peptoids against pathogenic and MDR strains (MIC, µM)

#		Pathogenic strains			MDR strains			
	*E. faecium* KCTC 13225	*E. faecalis* KCTC 3511	*P. aeruginosa* *^a^* KCTC 1637	*S. typhimurium* SL 1344	*P. aeruginosa* MDR‐198	*E. coli* ESBL 63103	*K. pneumonia* ESBL 61962	*S. aureus* MRSA C623
**1**	<0.8	0.8	6.3	25	6.3‐12.5	3.1‐6.3	6.3‐12.5	1.6‐3.1
**10**	12.5	12.5	nd^b^	12.5	nd^b)^	nd^b)^	nd^b)^	nd^b)^
**11**	<0.8	<0.8	3.1	25	6.3–12.5	3.1–6.3	6.3–12.5	3.1–6.3
**14**	<0.8	1.6	nd^b)^	12.5	nd^b)^	nd^b)^	nd^b)^	nd^b)^
**29**	1.6	3.1	3.1	6.3	6.3–12.5	≥25	>25	3.1–6.3
**32**	3.1	6.3	nd^b)^	6.3	6.3–12.5	12.5–25	12.5–25	3.1–6.3

All concentrations represented mean values of triplicates. *E. faecium*, *E. faecalis*, and *S. aureus* are gram‐positive, *P. aeruginosa*, *S. typhimurium*, *E. coli*, and *K. pneumonia* are gram‐negative strains.

^a)^ *P. aeruginosa* was maintained in refined LB media.

^b)^ Not determined. MDR: multi‐drug resistant, ESBL: extended spectrum *β*‐lactamase, MRSA: methicillin‐resistant *S. aureus*.

### 2.4 Conformational Analysis of Peptoids Under Membrane Mimicking Conditions Using CD Spectroscopy

**Figure 3:**
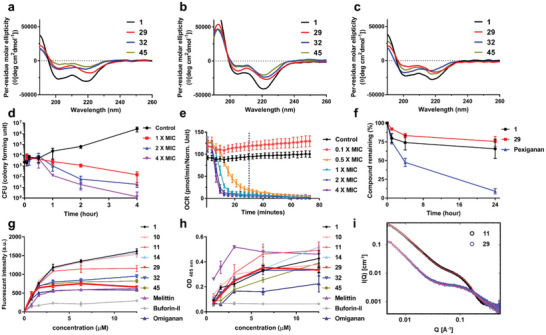
Conformational, biochemical, and mechanistic analysis of peptoids and AMPs. CD spectra of antimicrobial peptoids (50 µM) at 20 °C: a) in 5 mM Tris‐HCl buffer, b) in 5 mM lipid vesicles (POPE:POPG = 7:3 in molar ratio) in 10 mM Tris‐HCl buffer, c) in 5 mM lipid vesicles (POPC:cholesterol = 1:1 in molar ratio) in 10 mM Tris‐HCl buffer. d) Killing Kinetics. Time – killing study of *E. coli* ATCC 25922 challenged with various concentrations of **29**. e) The real‐time oxygen consumption rate (OCR, expressed in picomoles of molecular oxygen per minute) of *E. coli* ATCC 25922 treated with various concentrations of **29**. Dotted line indicates 30 min. f) Stability of peptoids (**1** and **29**) and pexiganan monitored after treatment with the human liver S9 fraction. g) Effect of antimicrobial peptides/peptoids on bacterial outer membrane tested with the NPN uptake assay in *E. coli* ATCC 25922. h) Effect of antimicrobial peptides/peptoids on bacterial inner membrane using an ONPG hydrolysis assay in *E. coli* ATCC 25922. i) SAXS data of peptoids **11** and **29** revealing distinct solution self‐assembled structures. SAXS data was collected in an aqueous environment at 5 mg mL^−1^ and plotted together with a best fit model (red line). Q is the scattering vector as defined in the experimental section. The y‐axis indicates scattering intensity (I).

To determine the conformation of peptoids in solution, circular dichroism (CD) spectroscopic analysis was performed. In Tris buffer, the CD spectra of peptoids **1**, **29**, **32**, and **45** showed the typical signature of a right‐handed polyproline type‐I (PPI) peptoid helix,^[^ ^35^ ^]^ with two CD minima at 202 and 220 nm (**Figure** **3**a). The fully helical peptoid **1** showed the strongest CD intensity, while the moderately helical peptoids **29**, **32**, and **45** exhibited weakened CD signals. CD spectra of peptoids were also recorded in the presence of lipid vesicles mimicking the membrane composition of bacteria or erythrocytes (Figure 3b,c, respectively). Under conditions mimicking bacterial cell membranes (POPE/POPG = 7:3), peptoids showed stronger peak at 220 nm, suggesting favorable interactions between the net anionic charge of the bacterial membrane and the cationic peptoids (Figure S13, Supporting Information).

### 2.5 Peptoid Self‐Assembly Structure in Aqueous Environment

To further characterize the structure of antimicrobial peptoids in solution, they were analyzed using SAXS. The self‐assembly structures of peptoids **11** and **29** were determined by SAXS in an aqueous environment followed by fit analysis. Although peptoids **11** and **29** only differ in a single monomer, where the hydrophobic *N*spe was substituted with a cationic *N*Lys, the toxicity of peptoid **29** against red blood cells showed dramatic reduction. Comparing the scattering plots for the two peptoids identified significant differences, with the plot of **11** showing a higher intensity than that of **29** at the same concentration (Figure 3i). Since the scattering length densities of these peptoids were similar, the observed difference in the intensity, together with the slope of the curve at high Q, indicated that **11** had the tendency to self‐assemble into larger defined nanostructures, while **29** remained monomeric and unstructured. This initial conclusion was confirmed by the fit analysis of the scattering data. The data for **29** could be explained by a polymeric random Gaussian chain model (radius of gyration of 10 Å). To explain the sharp upturn at low Q, the contribution of a small fraction (0.003–0.008 depending on concentration as shown in the supplementary information, Figure S15, Supporting Information) of big clusters had to be included in the analyzed model. The same model could not explain the observed peptoid **11** data. A cylindrical *α*‐helical bundle model, combining trimers (60% at 5 mg mL^−1^), dimers (10% at 5 mg mL^−1^), and monomers (30% at 5 mg mL^−1^), was compatible with the observations. This structure of peptoid **11** is similar to that recently reported for peptoid **1**.^[^ ^15b^ ^]^

### 2.6 Kinetics of Antibacterial Activity, Metabolic Stability, and Membrane Disruption Caused by Peptoids

To validate the antimicrobial activity of peptoid **29**, we performed bacterial growth inhibition and respiration assays. A dose‐ and time‐dependent bactericidal activity was seen in these experiments (Figure 3d). Bacteria were completely eradicated after 4 h when treated with **29** (4 × MIC). To monitor physiological changes in real‐time prokaryotic respiration assays were carried out by measuring the oxygen consumption rate (OCR) (Figure 3e). The OCR of **29**‐treated *E. coli* converged to zero after 30 min (marked by dotted line in Figure 3e), indicating the rapid growth inhibition when this peptoid was used at MIC concentration. More than 0.25 × MIC of **29** was required to induce a deceleration of OCR of *E. coli* (Figure S12a, Supporting Information). As shown in Figure S12b, Supporting Information, the addition of peptoid **29** resulted in an altered bacterial respiration pattern with a shape distinct from those seen after the used of bactericidal (e.g., ampicillin) and bacteriostatic (e.g., chloramphenicol) antibiotics.^[^ ^36^ ^]^ These findings indicate that peptoid **29** abolished oxygen consumption via a different mechanism than antibiotics inhibiting bacterial cell wall synthesis (e.g., ampicillin) or protein synthesis (e.g., chloramphenicol).

Peptoids have a nonnatural *N*‐alkylated amide backbone and are reported to be resistant to proteolytic degradation.^[^ ^37^ ^]^ We assessed the stability of **29** by exposing it to the human liver S9 fraction containing the metabolic enzyme pool. In these assays, peptoid **29** and the control peptoid **1** showed superior stability compared to the peptide‐based pexiganan during a 24 h incubation (Figure 3f).^[^ ^38^ ^]^

Next, we investigated how peptoids interacted with the bacterial membrane. We performed *N*‐phenyl‐1‐naphtylamin (NPN) uptake assays monitoring the disruption of the outer membrane and *o*‐nitrophenyl‐*β*‐D‐galactopyranosidase (ONPG) hydrolysis assays detecting damage to the cytoplasmic membrane (Figure 3g,h). In these experiments, melittin, omiganan, and peptoid **1** were used as positive controls and buforin‐II acted as a negative control. Peptoids with higher hydrophobicity (**1**, **10**, **11**, and **14**) strongly disrupted both the outer and inner membranes. Outer membrane disruption caused by the selective peptoids **29** and **32** was somewhat lower, comparable to the effect of omiganan and melittin. The degree of inner membrane disruption was also lower than that seen with more hydrophobic peptoids. The results indicated that decreased hydrophobicity, increased cationic charge, and the incorporation of the WW‐motif in **29** and **32** led to a reduction into their ability to disrupt bacterial membranes, especially the inner membrane.

To gain a detailed picture of peptoid‐cell membrane interactions, we also used SAXS analysis. This experiment provided insight into the preferred location of peptoids within the lipid bilayer. It has previously been shown that cationic amphipathic peptoids induced changes in liposomes, and the insertion of peptoids could be determined by a combination of SAXS and theoretical modeling.^[^ ^32^ ^]^ To investigate in detail the penetration of the cytoplasmic membrane by peptoids **11** and **29**, the peptoids where mixed with liposomes with a charge density mimicking that of *E. coli* (25% negative charged lipids). The data presented in Figure S14, Supporting Information, reveal that peptoids **11** and **29** interacted strongly with the lipid vesicles. A shift in the first minima towards higher Q upon increasing peptoid:lipid ratios clearly demonstrated this interaction (Figure S14a,c). This shift was more pronounced during interactions with peptoid **11**. In agreement with the results of the NPN and ONPG assays, peptoid **29** showed a less pronounced membrane interaction. Through the analysis, we found that peptoid **29** showed a broader distribution throughout the membrane and spanned the entire membrane, including the inner leaflet (Figure S14b,d, Supporting Information). In contrast, peptoid **11** seemed to interact more strongly with the outer leaflet. However, ≈96% of peptoid **11** was inserted into the hydrocarbon core of the membrane assuming a Gaussian distribution.

The presence of peptoids caused a significant thinning of the membrane. The thickness changed from 33.0 ± 1 Å to 30 ± 1 Å upon the addition of peptoid **11** and was reduced to 29 ± 1 Å in the presence of **29**. This finding is at variance with previous observations studying natural peptides,^[^ ^32a^ ^]^ where indolicidin only caused detectable membrane thinning at peptide‐to‐lipid ratios exceeding 1:10. This observation may indicate that both **11** and **29** significantly disrupted lipid packing upon insertion into the membrane. The broader distribution of **29** also suggests that this peptoid can cross the membrane more easily, potentially explaining its ability to reach intracellular targets as found in the following ODT imaging results.

### 2.7 Real‐Time Label‐Free Monitoring of *E. coli* Treated with Peptoid 29

**Figure 4:**
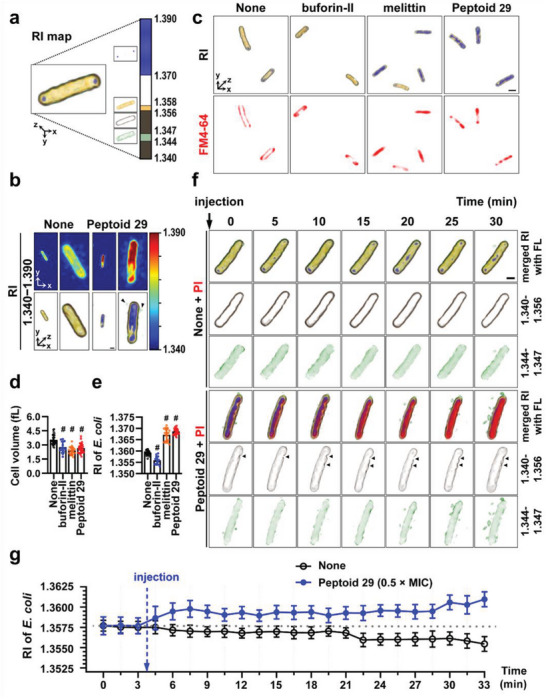
Effects of peptoid **29** on the membrane of *E. coli* ATCC 25922. a) False‐color reconstructed image of *E. coli* based on RI‐dependent segmentation masks. The color bar indicates 3D‐rendered RI distribution range (from 1.340 to 1.390). b) 2D and 3D‐reconstructed images with the RI distribution of untreated or **29**‐treated (4 × MIC) *E. coli* at 30 min. c) Representative 2D fluorescence and 3D RI distribution images of untreated (control), buforin‐II (25 µM), melittin (25 µM), or **29** (4 × MIC**)‐**treated *E. coli* with FM 4–64 (10 µg mL^−1^) at 30 min. Quantitative analysis of d) cell volume and e) mean RI for each group: none (*n* = 29), buforin‐II (*n* = 13), melittin (*n* = 19), and **29** (*n* = 28). Comparison of different groups was performed using unpaired and two‐tailed *t*‐test. # means *p* < 0.001. All values were presented as mean ± SD. f) Representative time‐lapse 3D‐rendered images of untreated or **29** (4 × MIC)‐treated *E. coli* shown at 5 min intervals over 30 min. Images were overlaid with corresponding fluorescence images treated with propidium iodide (PI) (10 µg mL^−1^). Black arrowheads point at sites of membrane damage. Scale bar: 1 µm. g) Quantitative analysis of time‐lapse monitoring for mean RI values of control or **29** (0.5 × MIC)‐treated *E. coli* at 1.5 min intervals over 30 min. Sample numbers were: control (*n* = 22) and **29** (*n* = 13).

To visualize the physical mechanisms underlying the bactericidal effect of peptoid **29** in living bacteria, we captured real‐time ODT images reconstructed by RI mapping (**Figure** **4**). We first analyzed morphological changes induced by treatment with two representative AMPs, melittin, and buforin‐II (Figure 1). These preliminary experiments established how these AMPs, with known mechanisms of action, affected RI distribution patterns. After this validation, we proceeded to use this imaging technique to investigate the mechanism of bacterial killing induced by peptoid **29** exposure.

As shown in Figure 4a, untreated *E. coli* was represented on 3D ODT images with RI values ranging from 1.340 to 1.390 in a single cell. We divided the RI map into four parts indicating bacterial membranes and intracellular regions. The yellow region (1.356–1.358) and brown region (1.340–1.356) indicate the intracellular domain and the bacterial membrane, respectively. The green region (1.344–1.347) shows peripheral cytoplasm adjacent to the membrane, and the blue region (1.370–1.390) represents structures with particularly high RI values.

ODT images of *E. coli* treated with **29** for 30 min were also acquired (Figure 4b). Compared to untreated *E. coli*, the 2D and 3D ODT images of bacteria treated with **29** depicted the intracellular region with a particularly high refractive index, suggestive of the aggregation of the intracellular components. Debris, cytoplasmic content leaking out into the extracellular space, was clearly visible around the disintegrating bacterial cell and was rendered in green (Figure 4b, see black arrowheads). This leakage of cellular content is reminiscent of the effects of membrane lysis observed in melittin‐treated *E. coli* (see Figure 1).

To substantiate a mechanism of membrane disruption, a lipophilic fluorescent dye, FM 4–64, was used for membrane staining. Bacterial membrane morphology after 30 min of treatment was observed both by fluorescence and ODT (Figure 4c). The effects of two peptide controls, melittin, and buforin‐II, were compared to peptoid **29**. In the absence of antimicrobials, bacteria exhibited an intact membrane structure that were elliptical and convex in shape. However, major changes in the internal and external morphology were observed in melittin and peptoid **29** treated *E. coli*, while the surface morphology of buforin‐II treated cells remained similar to that of the control cells. These findings demonstrated that peptoid **29** caused membrane disruption that showed striking similarity to the effects of melittin treatment. In melittin‐ and peptoid **29**‐treated *E. coli*, intracellular RI values increased (the blue region, 1.370–1.390) on ODT images, suggesting the aggregation of intracellular components.

Cell volumes and mean RI values were quantitatively analyzed in each treatment group. As summarized in Figure 4d,e, 30 min treatment with either buforin‐II, melittin, or **29** resulted in a notable reduction in cell volume. These observations are in line with previous reports suggesting that the inhibition of bacterial cell growth by AMPs led to the shrinkage of cells.^[^ ^39^ ^]^ When bacteria were treated with mellitin, buforin‐II, or peptoid **29**, the interruption of cell growth occurred within an hour, resulting in rapid cell death and concomitant decrease in cell volume. While changes in cell volume were uniform, irrespective of the antimicrobials used, changes in mean RI values showed characteristic differences depending on whether bacteria were treated with membrane permeating or membrane disrupting AMPs or peptoid (Figure 4e). After 30 min, buforin‐II treated *E. coli* showed decreased mean RI values compared to control cells. In contrast, both melittin and peptoid **29** significantly increased mean RI values suggesting that melittin and peptoid **29** utilized a similar mechanism in their antimicrobial action. Given that the buforin‐II‐mediated killing of bacteria in known to occur via the disintegration of the nucleoid, it appears that quantifying mean RI values can determine whether the antibacterial action of a peptoid is due to the targeting of intracellular components or the disruption of the cell membrane.

Next, we analyzed 3D reconstructed RI images obtained every 5 min over a 30 min period (Figure 4f). In these experiments, fluorescence caused by the intracellular entry of PI, a membrane‐impermeable dye intercalating and staining nucleoids, was simultaneously monitored. The detected PI fluorescence was overlaid with RI images. As demonstrated by the merged images, untreated *E. coli* maintained its RI distribution patterns over the observation period, while lack of PI fluorescence confirmed the intact state of *E. coli* cells. The segregated images based on RI maps showed a well‐defined cell membrane and retained cytoplasmic morphology (2nd row as a brown region, RI = 1.340–1.356 and 3rd rows as a green region, RI = 1.344–1.347) based on the specific RI values in the RI map. In contrast, *E. coli* treated with peptoid **29** clearly showed time‐dependent increases in PI fluorescence on the merged images, clearly demonstrating membrane damage and the consequent intracellular entry of PI. The RI images in the second row showed visible points of membrane disruption indicated by black arrowheads. In addition, the ongoing accumulation of leaking intracellular content is also clearly visible in the extracellular space.

To estimate the time needed for peptoid **29** to kill bacterial cells, we used the time‐lapse monitoring of mean RI values (Figure 4g). In these experiments, peptoid **29** was used at a lower concentration (0.5 × MIC) to slow down the rate of changes (see Figures S8 and S9 in supporting information for data obtained at various concentrations). This strategy was based on observations showing a slower decline in bacterial oxygen consumption rate at this peptoid concentration (Figure 3e). The mean RI values of *E. coli* were recorded every 1.5 min over 30 min. After the injection of peptoid **29** (after the third time point), the mean RI value of *E. coli* started to increase in a few minutes, while the mean RI values of untreated *E. coli* remained mostly unchanged. The slight decrease in RI values in the untreated control after 21 min was likely an artifact caused by the doubling of untreated *E. coli* cells.

Combined, these results indicated that peptoid **29** showed antimicrobial activity based on membrane disruption, as evidenced by the membrane leakage assays (Figure 3g,h) and by PI fluorescence and ODT images (Figure 4c,f). In addition, intracellular alterations also occurred after peptoid **29** treatment, indicated by real‐time live cell monitoring. The rapid increase in mean RI values in the intracellular region after peptoid **29** exposure suggested the rapid intracellular biomass flocculation as a second important antimicrobial mechanism in the action of this peptoid.

### 2.8 Intracellular Protein and Nucleic Acid Aggregation Caused by Peptoid 29

**Figure 5:**
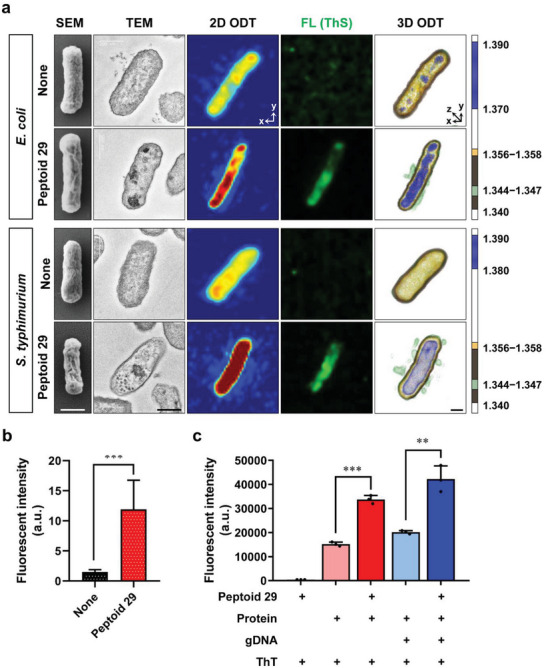
Effects of peptoid **29** on the morphology of gram‐negative bacteria. a) Scanning electron micrographs, transmission electron micrographs, RI‐based 2D projection images, fluorescence images using thioflavin S (Th S), and RI‐based 3D rendered images of *E. coli* (ATCC 25922) and *S. typhimurium* (SL 1344). Untreated and peptoid **29** (4 × MIC) treated cells. b) Quantitative analysis of Th S fluorescent intensity of control and peptoid **29** treated *E. coli*. c) In vitro aggregation assay of protein (500 µg mL^−1^) and/or gDNA (50 ng mL^−1^) in the presence or absence of **29** (25 µM), using thioflavin T (Th T) to detect the aggregation of macromolecules.

To gain additional insight into the antimicrobial action of **29**, morphological changes caused by the compound were observed using SEM, TEM, and ODT imaging (**Figure** **5**a). For comparison, we also tested another gram‐negative strain, *S. typhimurium*, in these experiments. As shown in Tables 2 and 3, peptoid **29** exhibited potent antimicrobial activity against both of these strains with identical MIC values of 6.3 µM. The SEM image of *E. coli* treated with peptoid **29** showed a roughly ridged surface, while the surface of untreated *E. coli* cells was finely textured. A similar change of surface morphology was observed in *S. typhimurium*, where treatment with **29** replaced the relatively smooth initial surface with one covered with thick wrinkles. Changes in intracellular morphology, visualized by TEM imaging, included the appearance of dark dots and clusters inside the bacteria after peptoid treatment, indicating the aggregation of intracellular components.

To verify the intracellular aggregation caused by peptoid **29** involves proteins and nucleic acids, we performed fluorescence imaging using thioflavin S (Th S). Thioflavins are widely used fluorescent dyes that visualize the aggregation of proteins (e.g., amyloids) and nucleic acids.^[^ ^40^ ^]^ Initially, we attempted to use thioflavin T (Th T), but the background signal prevented the simultaneous analysis of the cells using our ODT instrument. There was no fluorescent signal in the intracellular space of untreated *E. coli* and *S. typhimurium* cells after Th S staining. However, after treatment with peptoid **29**, the aggregated biomass of proteins and nucleic acids led to the enhanced green fluorescence of Th S (Figure 5a fourth column). Quantifying the average fluorescent intensity indicated a significantly increased emission after peptoid **29** treatment (Figure 5b). Similarly, enhanced Th S fluorescence was observed for *E. coli* treated with melittin and peptoid 1, whereas buforin‐II did not show evidence of nonspecific intracellular biomass aggregation. (Figure S11, Supporting Information).

To demonstrate that the observed increase in Th S fluorescence was the result of the aggregation of intracellular macromolecules, we conducted in vitro experiments to investigate whether peptoid **29** could cause the aggregation of proteins and/or nucleic acids. In these experiments, Th T was used as a fluorescent marker of macromolecule aggregation. In the absence of bacterial genomic DNA (gDNA) or proteins, peptoid **29** alone induced negligible Th T fluorescence monitored at *λ*_ex_ = 450 nm and *λ*_em_ = 485 nm (Figure 5c). At this excitation wavelength, autofluorescence from the protein and gDNA mixture was also minimal (data not shown). In the absence of peptoid **29**, samples containing proteins and Th T exhibited noticeable fluorescence emission and the addition of gDNA to this mixture led to a further increment in fluorescence intensity. However, in the presence of peptoid **29**, both samples (Th T/protein and Th T/protein/DNA) showed significant increases in fluorescent emission, with the sample containing both protein and gDNA exhibiting the highest fluorescence intensity. It should be noted that in these in vitro experiments reaching a protein or DNA concentration seen in the intracellular space of live bacterial cell is not trivial. Therefore, dilute concentrations of gDNA (50 ng mL^−1^) and protein extracts (500 µg mL^−1^) were used in this assay. The actual cytoplasmic concentrations of nucleic acids are>100 mg mL^−1^ (i.e., DNA ≈10 mg mL^−1^ and RNA ≈100 mg mL^−1^) while the protein concentration is >200 mg mL^−1^.^[^ ^41^ ^]^ Therefore, aggregation caused by peptoid **29** in the bacterial cytoplasm is likely to occur more rapidly and would be more striking. From this in vitro investigation, we could conclude the following: 1) among the components of intracellular biomass, proteins alone could form aggregates with peptoid **29**; and 2) it is likely that the anionic gDNA and ribosomes could interact with the cationic amphipathic peptoid **29** based on electrostatic and hydrophobic interactions. Taken together, we propose that peptoid **29** caused nonspecific intracellular biomass aggregation leading to rapid bacterial death.

## 3 Conclusion

In summary, we verified 3D ODT imaging as a useful technique for investigating the mechanisms of action of antimicrobial peptides and peptoids. In contrast to EM imaging techniques, using ODT enables the real‐time monitoring of morphological changes occurring in the extracellular space and inside the bacterial cells without the use of any fluorescent labels. Morphological analysis of gram‐negative bacteria using 3D ODT with specific RI distributions is a promising technique to verify the antimicrobial mechanisms of various AMPs and peptidomimetics. In addition, ODT monitoring may facilitate the rapid screening of antibiotic agents through time‐lapse monitoring of RI values without extra pretreatment steps.

**Figure 6:**
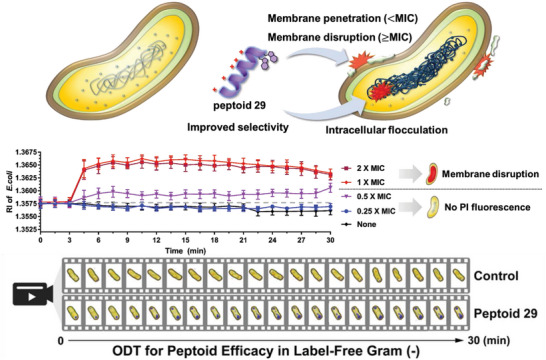
Illustration of the expected multitarget antimicrobial mechanisms of peptoid **29**. Monitored RI value changes depending on the concentration of **29**. 3D ODT images of **29** (0.5 × MIC)‐treated *E. coli* were recorded over 30 min during label‐free real‐time monitoring (a movie file is uploaded in the supporting information).

In our discovery program, screening of an indole‐containing peptoid library provided peptoid **29** as a hit compound. SAR analysis demonstrated the importance of WW‐motif and optimal cationic charge‐to‐length ratio for potent antimicrobial activity and improved selectivity. Mechanistic studies, comparing peptoid **29** to well‐known AMPs, melittin, and buforin‐II, demonstrated that the mechanism of action of peptoid **29** is more similar to melittin than buforin‐II, indicating that peptoid **29** is a membrane disrupting compound. At 1.0 × MIC or higher concentrations (e.g., 2.0 × MIC), membrane disruption was followed by peptoid internalization leading to rapid intracellular biomass aggregation. (Figures S8 and S9, Supporting Information) This latter phenomenon was similar to the action of buforin II. At low concentrations (e.g., 0.5 × MIC), peptoid **29** induced an increase of intracellular RI values over time (Figure 4g) without any apparent disruption of bacterial membrane (**Figure** **6**). Notably, a rapid decrease in metabolic activity at more than 0.3 × MIC peptoid concentrations was detected by bacterial respiration assays in *E. coli* (Figure 3e and S12a, Supporting Information). In our previous report, cationic amphipathic peptoids, such as peptoid **1**, were shown to exhibit intrinsic cell‐penetrating properties.^[^ ^42^ ^]^ It has been suggested that accumulation of AMPs in the bacterial membrane causes increased permeability due to the loss of membrane integrity (e.g., destabilization of the LPS layer).^[^ ^43^ ^]^ With peptoid **29**, the mechanism leading to the internalization of the compound at low concentrations is not fully understood. However, the observed inhibition of bacterial metabolism and the increased intracellular RI values with fluorescent images proves peptoid internalization in the absence of visible pore formation at low peptoid concentrations. The rapid increment of RI values caused by inflow of peptoid through the membrane disruption was also confirmed. It is identified that the peptoid **29** shows intracellular flocculation basically, and different behaviors to bacterial membrane depending on its own concentrations.

Brogden noted previously that the membrane disruption is not the only mechanism in the antibacterial action of AMPs. Their ability to function as metabolic inhibitors was also a major potential mode of action that must not be overlooked.^[^ ^4b^ ^]^ The compound described here, peptoid **29**, kills bacteria via a multitarget mechanism, causing bacterial cell death by disrupting/penetrating the cell membrane and aggregating intracellular proteins and nucleic acids, eventually causing rapid cell death. Further in‐depth studies are needed to clarify further the sequence of events during peptoid action, its effects on different bacterial components, and the possible difference in antibacterial mechanisms depending on peptoid concentrations.

At this point, it is not clear whether membrane disruption and depolarization is the primary cause of bacterial death, or if the aggregation of intracellular biomass is a key mechanism in the antibacterial action of peptoid **29**. We suggest that potentially both mechanisms act simultaneously and synergistically. As these events occur rapidly, within a few minutes of the addition of the peptoid, it is technically challenging to capture changes in bacterial morphology within a sufficiently narrow time window to derive definitive answers. Ongoing work to further refine the real‐time monitoring of bacterial morphology using ODT may help answering these questions. In addition, a better understanding of the antibacterial mechanisms of peptoids will help designing antimicrobial compound that strike multiple bacterial targets precisely, without damaging host cells. Such advances will increase the possibility of overcoming bacterial resistance and will lead to successful clinical applications.

## 4 Experimental Section

### Materials

Reagents and solvents were purchased from commercial vendors and used without further purification. Rink amide MBHA resin, 2‐chlorotrityl resin, and Fmoc‐protected amino acids were purchased from Merck Millipore (Billerica, MA, USA). Trifluoroacetic acid (TFA) and *N*,*N*‐dimethylformamide (DMF, >99.9%, peptide synthesis grade) were purchased from Acros Organics (Fair Lawn, NJ, USA). Acetonitrile (ACN, HPLC grade), propidium iodide (PI), and FM4‐64 were purchased from Thermo Fisher Scientific (Waltham, MA, USA). *N*,*N’‐*Diisopropylcarbodiimide (DIC) was purchased from Chem‐Impex (Wood Dale, IL, USA). 1‐Palmitoyl‐2‐oleoyl‐*sn*‐*glycero*‐3‐phosphoethanolamine (POPE), 1‐palmitoyl‐2‐oleoyl‐*sn*‐*glycero*‐3‐phosphocholine (POPC), 1‐palmitoyl‐2‐oleoyl‐*sn*‐*glycero*‐3‐phospho‐(1’‐*rac*‐glycerol) (POPG), 2‐dimyristoyl‐*sn*‐*glycero*‐3‐phosphocholine (DMPC), 1,2‐dimyristoyl‐*sn*‐*glycero*‐3‐phospho‐(10‐*rac*‐glycerol) (DMPG), and 1,2‐dimyristoyl‐*sn*‐*glycero*‐3‐phosphoethanolamine‐*N*‐[methoxy(polyethylene glycol)‐2000] (DMPE‐PEG) were purchased from Avanti Polar Lipids (Alabaster, AL, USA). Mueller‐Hinton broth 2 (MHB2), bovine serum albumin (BSA), thioflavin T (Th T), thioflavin S (Th S), acetic acid, poly‐L‐lysine solution, and human liver S9 were purchased from Sigma‐Aldrich (St. Louis, MO, USA). Other chemical reagents and solvents were purchased from TCI (Tokyo, Japan) and Alfa Aesar (Haverhill, MA, USA).

### Synthesis of Peptoids

Control peptides (melittin, buforin‐II, pexiganan, and omiganan) were synthesized according to the standard solid‐phase peptide synthesis (SPPS) protocol using 9‐fluorenylmethoxycarbonyl (Fmoc) chemistry. Peptide sequences were synthesized on a Fmoc‐Rink amide MBHA resin (0.59 mmol g^−1^) using a Tribute peptide synthesizer (Gyros Protein Technologies, Tucson, AZ, USA). The coupling procedure used 3 equivalents of Fmoc‐L‐amino acid, 3 equivalents of HATU (1‐[bis(dimethylamino)methylene]‐1*H*‐1,2,3‐triazolo[4,5‐b]pyridinium 3‐oxide hexafluorophosphate), and *N*‐diisopropylethylamine (DIEA) at 8 equivalents in DMF at room temperature for 1 h. The Fmoc deprotection was carried out by treating the resin with 20% (v/v) piperidine in DMF for 5 min. After completion of the synthesis, the peptide was cleaved from the resin with a mixture of 95% TFA, 2.5% water, and 2.5% triisopropylsilane for 2 h. Peptoids were synthesized according to the solid‐phase submonomer protocol.^[^ ^44^ ^]^ Reactions were carried out in a solid‐phase extraction cartridge assembled with a polyethylene filter (Applied Separations, Allentown, PA, USA). The reactions were accelerated by microwave irradiation using a CEM MARS multimodal microwave reactor equipped with a fiber‐optic temperature probe and a magnetic stirrer (CEM Corp., Matthews, NC, USA). Peptoid sequences were synthesized manually on a Fmoc‐Rink amide MBHA resin (0.59 mmol g^−1^). Fmoc deprotection was performed by treatment with 20% (v/v) piperidine in DMF twice at 80 °C (600 W max power, ramp 2 min, hold 2 min, stirring level 2). Typically, a reaction scale of 0.065 mmol was used (0.10 g of resin). The resin was washed sequentially with the following: CH_2_Cl_2_ (×3), DMF (×3), CH_3_OH (×1), DMF (×3), and CH_2_Cl_2_ (×3). Bromoacetylation was carried out on the deprotected resin using bromoacetic acid (1.2 M in DMF, 20 equiv.) and DIC (20 equiv.) at 35 °C (300 W max power, ramp 30 s, hold 1 min, stirring level 2). The resin was washed in the same sequence as described above. Amine submonomers were then incorporated by an S_N_2 reaction at 95 °C (300 W max power, ramp 2 min, hold 90 s, stirring level 2). Bromoacetylation and displacement with the amine submonomer were repeated until the desired peptoid sequence was obtained. For amine submonomers, *N*Trp(Boc), *N*Lys(Boc), *N*4hb(TIPS), benzylamine (*N*pm), or (*S*)‐1‐phenylethylamine (*N*spe) were used as a solution dissolved in *N*‐methyl‐2‐pyrrolidone (NMP) (1.0 M, 20 equiv.). Cleavage from the resin was achieved using a cleavage solution (TFA: CH_2_Cl_2_:triisopropylsilane = 95:2.5:2.5 [v/v/v]) for 10 min at room temperature.

### Antibacterial Activity Assay

The antibacterial activity of the peptoids was evaluated against gram‐positive (*Staphylococcus aureus*, ATCC 25 923) and gram‐negative (*Escherichia coli*, ATCC 25 922) bacteria. Antibacterial activity was reported as the minimum inhibitory concentration (MIC), which is the lowest concentration of the antibacterial agent that inhibits a microorganism's growth after overnight incubation at 37 °C. The MIC of each peptoid was determined using a broth dilution assay. For each assay, the primary culture of either *S. aureus* or *E. coli* was grown overnight in cation‐adjusted Mueller Hinton broth 2 (MHB2) media at 37 °C with shaking. A secondary culture was prepared the next day, grown for 3–4 h, and then diluted in MHB2 media with a final concentration of 0.001% acetic acid and 0.02% bovine serum albumin to obtain a calculated optical density (OD) of 0.001. This corresponded to ≈2–5 × 10^5^ colony‐forming units (CFU mL^−1^) of bacteria. Using 96‐well polypropylene plates (Eppendorf, Hamburg, Germany), 100 µL of 2–5 × 10^5^ CFU mL^−1^ bacteria were added in triplicate for each peptoid. Each peptoid was assayed at six different concentrations in two‐fold serial dilutions. The maximum peptoid concentration was 25 µM. The plate was then incubated at 37 °C for 24 h, and MIC data were recorded by measuring the OD value at 600 nm on a microplate reader (BioTek Instrument, VT, USA). The MIC value determination was performed in triplicate. The MIC was defined as the lowest peptoid concentration that prevented turbidity during visual inspection.

### Hemolysis Assay

The hemolysis assay was performed using rat erythrocytes in phosphate‐buffered saline (PBS), as reported by Mendes et al.^[^ ^45^ ^]^ Animal studies were approved by the Laboratory Animal Resource Center of Gwangju Institute of Science and Technology. Blood was collected from a 13‐week‐old male Sprague‐Dawley rat in tubes containing 158 USP units of sodium heparin to prevent coagulation and centrifuged at 1000 rpm for 10 min at 4 °C. Plasma was removed carefully and erythrocyte pellets were washed three times with PBS (35 mM phosphate, pH 7.4, 150 mM NaCl). Next, 150 µL of a 10% erythrocyte suspension was mixed with 50 µL of serially diluted peptoids (0–100 µM) in a 96‐well black polypropylene plates (Greiner, Kremsmünster, Austria). Untreated erythrocytes and cells lysed by the addition of 1% Triton X‐100 were used as negative and positive controls, respectively, with the 1% Triton X‐100‐treated wells being accepted to represent 100% hemolysis. The reaction mixture was incubated at 37 °C for 60 min, and the plates were centrifuged at 1000 rpm for 15 min. Aliquots (150 µL) of the supernatant were transferred to fresh 96‐well plates, and the absorbance of each well was measured at 540 nm using a microplate reader. Hemolysis induced by peptoids was calculated as the percentage value of Triton X‐100 lysed cells. The percentage of hemolysis by the extracts was calculated according to the following formula: % hemolysis = [(Abs 540 nm in the peptoid solution – Abs 540 nm in PBS) / (Abs 540 nm in 1% Triton X‐100 – Abs 540 nm in PBS)] × 100. HC_10_ and HC_50_ were defined as the peptoid concentrations that caused 10% and 50% hemolysis in rat erythrocytes, respectively. *H*_max_ indicates the percentage of hemolysis observed at the maximum peptoid concentration, 100 µM throughout this study. All tests were conducted in triplicate, and the error between these was <10%.

### ODT and Fluorescence Imaging

Three‐dimensional (3D) optical diffraction tomography (ODT) and fluorescence images of live bacterial cells were obtained using an HT‐2H Mach−Zehnder interferometric microscope, combining both ODT and fluorescence imaging into a single unit (Tomocube, Daejon, South Korea). 3D Fluorescence imaging was carried out, using three light sources (385, 470, and 565 nm) via serial fluorescence image acquisition in multiple planes followed by 3D deconvolution. 3D correlative imaging, using a combination of ODT and fluorescence imaging was performed using commercial software (TomoStudioTM, Tomocube) as described previously^[^ ^23c^ ^]^ Bacterial cells were grown overnight and diluted (≈10^7^ CFU mL^−1^) in cation‐adjusted MHB2 media. The cells were loaded onto 50 mm imaging dishes with a #1.5H glass coverslip bottom (TomoDish, Tomocube) that were precoated with 0.01% (w/v) poly‐L‐lysine (Sigma‐Aldrich) in water. After 30 min incubation at room temperature, the coverslips were washed twice with PBS (pH 7.4) to remove unattached cells, and the media was replaced with PBS for the duration of the imaging. To monitor the change in bacterial morphology, bacterial cells were treated with a peptoid (or with control AMPs) in PBS containing FM4‐64 (10 µg mL^−1^) or propidium iodide (10 µg mL^−1^). To analyze 3D refractive index (RI) tomograms, individual bacteria were captured using ODT equipped with a digital micromirror device (Tomocube). In more detail, the light scattered by the bacterial cells was collected by an objective lens (NA = 1.2, water dipping, UPLSAPO 60XW, Olympus) and recorded by a digital image sensor (CMOS camera, FL3‐U313Y3M‐C, FLIR Systems). Using a field retrieval algorithm, the amplitude and phase images were retrieved from the obtained multiple holograms, and then the 3D RI distribution was determined.

### Quantitative Analysis of Bacterial Cell Parameters

The analysis tool in the TomoStudio software (Tomocube) was used to measure specific values, including the refractive index (RI) and the cell volume of bacteria. These values were determined from RI increments (RII) of 0.190 fL pg^−1^, which is known as a representative value and was assumed to be the same for most proteins.^[^ ^46^ ^]^

### Statistical Analysis

Statistical analysis was performed using GraphPad Prism 8 or the Origin 2018 program. Comparison of different groups was performed using unpaired and two‐tailed *t*‐tests. All values were presented as mean ± SD. *p < 0.05, **p < 0.01 and ***p < 0.001.

### SEM Imaging

Samples (*E. coli* ATCC 25 922 or *S. typhimurium* SL 1344 cells) were fixed with 2% glutaraldehyde and 2% paraformaldehyde in cacodylate buffer (50 mM, pH 7.4) for 1 h at 4 °C. The samples were dehydrated in a graded series of ethanol (50, 60, 70, 80, 90, and 100%) for 10 min each, then kept in a mixture of 100% ethanol and isoamyl acetate (2:1, 1:1, and 1:2) for 10 min, and finally in pure isoamyl aetate for 15 min. After the removal of isoamyl acetate, the samples were treated with hexamethyldisilazane (HMDS) for 40 min. After the removal of HMDS, samples were air‐dried for ≈1 h and then sputter‐coated with a thin layer of gold. Samples were viewed under a SUPRA 55VP scanning electron microscope (Carl Zeiss, Germany) at an accelerating voltage of 3 kV in Chuncheon center, Korea Basic Science Institute.

### TEM Imaging

Samples were fixed with 2% glutaraldehyde and 2% paraformaldehyde in cacodylate buffer (50 mM, pH 7.4) for 1 h at 4 °C and then postfixed with 2% osmium tetroxide and 3% potassium hexacynoferate for 40 min. The samples were dehydrated in a graded series of ethanol (50, 60, 70, 80, 90, and 100) for 10 min each, then kept in a mixture of 100% ethanol and LR white resin (2:1, 1:1, and 1:2) for 10 min, and finally in pure LR white resin for 15 min. Samples were transferred to a dry capsule or mold, and the mold was filled with embedding resin. The embedded samples were cured in a 60 °C oven for 24 h. Ultra‐thin sections (80 nm) were cut and placed on a copper grid. The final samples were stained with uranyl acetate and lead citrate and viewed under a JEM‐2100F transmission electron microscope (JEOL, Japan) at an accelerating voltage of 200 kV at Chuncheon Center, Korea Basic Science Institute.

### Small Angle X‐ray Scattering

Prior to the experiment, peptoids were dissolved in Tris buffer (50 mM, pH 7.4). This peptoid stock solution (5 mg mL^−1^) was diluted as needed. Unilamellar lipid vesicles were prepared using synthetic DMPC, DMPG, and DMPE‐PEG at a 75:22.5:2.5 molar ratio. The lipids were first dissolved in a 1:3 (v/v) methanol:chloroform solution. To make a lipid film, the organic solvents were removed completely under vacuum using a rotary evaporator with a Vacuubrand vacuum pump. The resulting lipid film was hydrated at 35 °C using Tris buffer (50 mM, pH 7.4). After hydration, the dispersion was sonicated for 15 min to promote the formation of smaller unilamellar vesicles. This was followed by the extrusion of lipid vesicles through a 100 nm pore diameter polycarbonate filter (>21 times) using an Avanti mini‐extruder fitted with two 1 mL airtight syringes. Immediately before the SAXS data collection, a peptoid solution with the adequate concentration for the target lipid:peptoid ratio was mixed 1:1 with the lipid solution (5 mg mL^−1^) using a micropipette. SAXS experiments were performed at the automated BM29 bioSAXS beamline at the European Synchrotron Radiation Facility (ESRF) in Grenoble, France.^[^ ^47^ ^]^ The data was obtained using an energy of 12.5 keV and a detector distance of 2.87 m, covering a Q range (the scattering vector *Q*  =  4*π* sin(*θ*/2)/*λ*), where 𝜃 is the scattering angle and 𝜆 is the X‐ray wavelength) of ≈0.0049 Å^−1^ to 0.5205 Å^−1^. All experiments were conducted at 37 °C. The data set was calibrated to an absolute intensity scale using water as a primary standard. Samples (45 µL) were run through a capillary using the flow mode of the automated sample changer.^[^ ^48^ ^]^ SAXS data was collected in ten successive frames of 0.5 s each to monitor radiation damage, and the data reduction was carried out using BioXTAS RAW program.^[^ ^49^ ^]^

### Theoretical Analysis of SAXS Data

All scattering models were implemented and fitted to the experimental SAXS data using the QtiSAS software.^[^ ^50^ ^]^ The SAXS results for peptoid **11** were analyzed using a theoretical cylindrical helical bundle model described in detail previously,^[^ ^51^ ^]^ while data for peptoid **29** were analyzed using a random polymer‐like chain model.^[^ ^32a^ ^]^

## Conflict of Interest

The authors declare no conflict of interest.

## Author Contributions

M.K., Y.C., and D.S contributed equally to this work., and each reserve the right to put their name first on their respective CVs.

## Supporting information

Supporting Information

Supplemental Movie 1

## Data Availability

The data that support the findings of this study are available in the supplementary material of this article.
