# Supplementary material for: Real‐Time Monitoring of Multitarget Antimicrobial Mechanisms of Peptoids Using Label‐Free Imaging with Optical Diffraction Tomography
Source: Adv Sci (Weinh). 2023 Jun 21;10(24):2302483. doi: 10.1002/advs.202302483 (PMC10460844; doi:10.1002/advs.202302483)
Supplement: Supplementary file 1 — Supporting Information [file ADVS-10-2302483-s002.pdf]

ADVANCED SCIENCE
Open Access

Supporting Information

for *Adv. Sci.*, DOI 10.1002/advs.202302483

Real-Time Monitoring of Multitarget Antimicrobial Mechanisms of Peptoids Using Label-Free Imaging with Optical Diffraction Tomography

Minsang Kim, Yeongmi Cheon, Dongmin Shin, Jieun Choi, Josefine Eilsø Nielsen, Myeong Seon Jeong, Ho Yeon Nam, Sung-Hak Kim, Reidar Lund, Håvard Jenssen, Annelise E. Barron, Seongsoo Lee and Jiwon Seo**

Supporting Information

Real-time monitoring of multi-target antimicrobial mechanisms of peptoids using label-free imaging with optical diffraction tomography

*Minsang Kim, Yeongmi Cheon, Dongmin Shin, Jieun Choi, Josefine Eilsø Nielsen, Myeong Seon Jeong, Ho Yeon Nam, Sung-Hak Kim, Reidar Lund, Håvard Jenssen, Annelise E. Barron, Seongsoo Lee** *and Jiwon Seo**

List of contents

1. Peptoid submonomers··················S3

2. Sequences, properties, and chemical structures of peptoids··················S4

3. Antimicrobial and hemolytic activity··················S10

4. Cell culture and cytotoxicity test using the MTS assay. ··················S12

5. Structure-activity relationship (SAR) analysis··················S12

6. Effect of counter-ion exchange on antimicrobial, hemolytic, and cytotoxic activity ·······S15

7. HPLC chromatogram, retention time (t_R), and LC-MS data ··················S19

8. Growth kinetics against *P. aeruginosa*··················S26

9. Real-time monitoring of *E. coli* treated with peptoid **29** using 3D optical diffraction tomography

…S27

10. Representative fluorescent images and RI-based 3D rendered images of *E. coli* treated with melittin, buforin-II, and peptoid **1** in the presence of thioflavin …S29

11. Bacterial respiration assay…S30

12. Circular dichroism spectra …S31

13. Small angle X-ray scattering (SAXS) data…S33

14. In vitro biological assays…S34

15. References…S36

List of Figures and Tables

Figure S1. Peptoid synthesis and monomer structures…S3
Figure S2. Chemical structures of peptoids **1**–**66**…S6–S9
Figure S3. Effect of the number of *N*Trp monomer(s) on the *E. coli* MIC value…S13
Figure S4. Quantitative analysis of ^{19}F-NMR after counter-ion exchange…S15
Figure S5. HPLC chromatograms of counter-ion exchanged peptoids with UV detection at 220 nm…S17
Figure S6. HPLC chromatograms of peptoids **1 - 66** with UV detection at 220 nm ··S20–S23
Figure S7. Effect of antimicrobial peptoids on bacterial growth kinetics …S26
Figure S8. Real-time monitoring of *E. coli* treated with **29** (1 × MIC) using 3D optical diffraction tomography…S27
Figure S9. Quantitative analysis of time-lapse monitoring for mean RI values of non-treated and different concentrations of **29** (0.25, 0.5, 1, 2 × MIC)-treated *E. coli* at 1.5 min intervals over 30 min. …S28
Figure S10. Quantitative analysis of time-lapse monitoring for a) volume, b) mass, and c) biomolecular density of control or **29** (0.5 × MIC)-treated *E. coli* at 1.5 min intervals over 30 min. …S28

Figure S11. Effects of melittin, buforin-II, and peptoid **1** treatment on the morphology of *E. coli* (ATCC 25922) and on the thioflavin fluorescence. ··S29
Figure S12. Real-time changes in oxygen consumption rate in response to treatment with peptoid **29**, ampicillin (Amp), and chloramphenicol (Cam) in *E. coli* measured on a Seahorse XFe 96 extracellular flux analyzer.··S30
Figure S13. CD spectra of peptoids in the three environments ·································S31
Figure S14. Membrane interaction of peptoids **11** and **29**···································· S33
Figure S15. SAXS data revealing the concentration dependence of the self-assembled structure of peptoids ·· S34
Table S1. Sequences and properties of antimicrobial peptoids **1 - 66**·······················S4–S5
Table S2. Antimicrobial and hemolytic activities of **1 − 66**······························S10–S11
Table S3. Antimicrobial, hemolytic, and cytotoxic activities of counter-ion-exchanged peptoids ···S18
Table S4. Electrospray ionization-mass spectrometry data of peptoids **1 - 66**··········S24–S26

1. Peptoid submonomers

Peptoids were synthesized according to the solid-phase submonomer protocol (**Figure S1**a). Five submonomers were used for the peptoid library synthesis (**Figure S1**b). To synthesize helical peptoids with right-handedness, the helix-inducing chiral submonomer (*S*)-(-)-1-phenylethylamine (*N*spe) was incorporated. Benzylamine (*N*pm) was used as an achiral aromatic submonomer. Tryptamine (*N*Trp) was used to introduce an indole side chain to the peptoid sequence. Cationic charge(s) were incorporated by using 1,4-diaminobutane (*N*Lys) as a submonomer. 4-Amino-1-butanol (*N*4hb) was used as a polar neutral side chain in control peptoids designed to investigate the role of the cationic *N*Lys. The protected submonomers, *N*Trp(Boc),[1] *N*Lys(Boc),[2] and *N*4hb(TIPS)[3] were prepared for peptoid synthesis according to previously reported procedures.

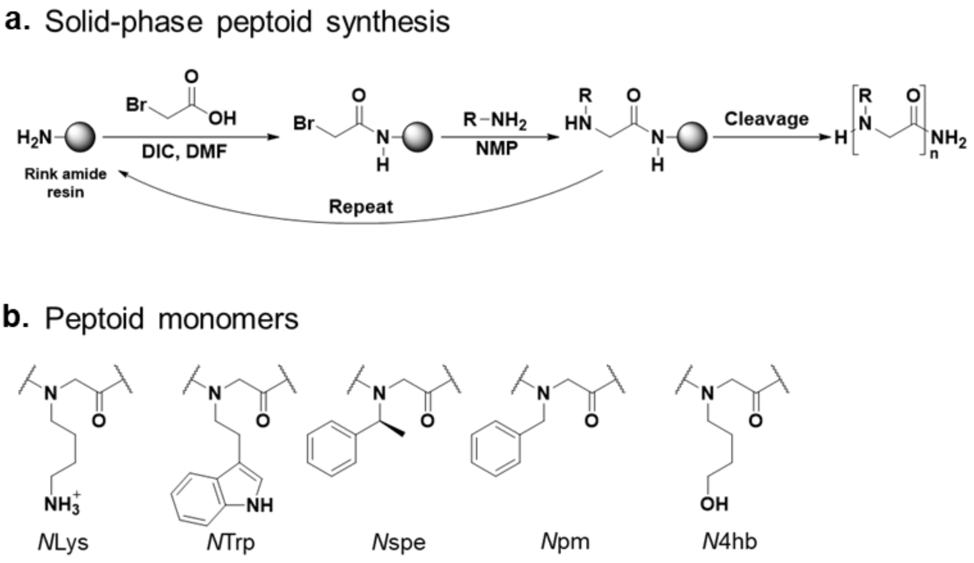

Figure S1. Peptoid synthesis and monomer structures

2. Sequences, properties, and chemical structures of peptoids

Table S1. Sequences and properties of antimicrobial peptoids **1 − 38**

Comp'd	Sequence	CTLR[a]	# of *N*Trp	HPLC elution[b]
**1**	H-**_N_Lys**-*N*spe-*N*spe-**_N_Lys**-*N*spe-*N*spe-**_N_Lys**-*N*spe-*N*spe-**_N_Lys**-*N*spe-*N*spe-NH_2	0.33	0	54.5
**2**	H-**_N_Lys**-*N*pm-*N*pm-**_N_Lys**-*N*pm-*N*pm-**_N_Lys**-*N*pm-*N*pm-**_N_Lys**-*N*pm-*N*pm-NH_2	0.33	0	50.7
**3**	H-*N*Trp-**_N_Lys**-*N*spe-*N*spe-**_N_Lys**-*N*spe-*N*spe-**_N_Lys**-*N*spe-*N*spe-**_N_Lys**-*N*spe-*N*spe-NH_2	0.31	1	55.8
**4**	H-**_N_Lys**-*N*spe-*N*spe-**_N_Lys**-*N*spe-*N*spe-**_N_Lys**-*N*spe-*N*spe-**_N_Lys**-*N*spe-*N*spe-*N*Trp-NH_2	0.31	1	57.0
**5**	H-*N*Trp-**_N_Lys**-*N*spe-*N*spe-**_N_Lys**-*N*spe-*N*spe-**_N_Lys**-*N*spe-*N*spe-**_N_Lys**-*N*spe-*N*spe-*N*Trp-NH_2	0.29	2	58.6
**6**	H-*N*Trp-*N*Trp-**_N_Lys**-*N*spe-*N*spe-**_N_Lys**-*N*spe-*N*spe-**_N_Lys**-*N*spe-*N*spe-**_N_Lys**-*N*spe-*N*spe-NH_2	0.29	2	57.2
**7**	H-**_N_Lys**-*N*spe-*N*spe-**_N_Lys**-*N*spe-*N*spe-**_N_Lys**-*N*spe-*N*spe-**_N_Lys**-*N*spe-*N*Trp-NH_2	0.33	1	54.1
**8**	H-**_N_Lys**-*N*spe-*N*spe-**_N_Lys**-*N*spe-*N*spe-**_N_Lys**-*N*spe-*N*Trp-**_N_Lys**-*N*spe-*N*spe-NH_2	0.33	1	54.5
**9**	H-**_N_Lys**-*N*spe-*N*spe-**_N_Lys**-*N*spe-*N*Trp-**_N_Lys**-*N*spe-*N*spe-**_N_Lys**-*N*spe-*N*spe-NH_2	0.33	1	54.6
**10**	H-**_N_Lys**-*N*spe-*N*Trp-**_N_Lys**-*N*spe-*N*spe-**_N_Lys**-*N*spe-*N*spe-**_N_Lys**-*N*spe-*N*spe-NH_2	0.33	1	54.9
**11**	H-**_N_Lys**-*N*spe-*N*spe-**_N_Lys**-*N*spe-*N*spe-**_N_Lys**-*N*spe-*N*spe-**_N_Lys**-*N*Trp-*N*Trp-NH_2	0.33	2	54.0
**12**	H-**_N_Lys**-*N*spe-*N*spe-**_N_Lys**-*N*spe-*N*spe-**_N_Lys**-*N*Trp-*N*Trp-**_N_Lys**-*N*spe-*N*spe-NH_2	0.33	2	55.9

**13**	H-**NLys**-Nspe-Nspe-**NLys**-NTrp-NTrp-**NLys**-Nspe-Nspe-**NLys**-Nspe-Nspe-NH_2	0.33	2	56.0
**14**	H-**NLys**-NTrp-NTrp-**NLys**-Nspe-Nspe-**NLys**-Nspe-Nspe-**NLys**-Nspe-Nspe-NH_2	0.33	2	55.6
**15**	H-**NLys**-Nspe-Nspe-**NLys**-Nspe-NTrp-**NLys**-NTrp-Nspe-**NLys**-Nspe-Nspe-NH_2	0.33	2	53.7
**16**	H-**NLys**-Nspe-NTrp-**NLys**-Nspe-Nspe-**NLys**-Nspe-Nspe-**NLys**-Nspe-NTrp-NH_2	0.33	2	54.7
**17**	H-**NLys**-NTrp-Nspe-**NLys**-Nspe-Nspe-**NLys**-Nspe-Nspe-**NLys**-Nspe-NTrp-NH_2	0.33	2	56.0
**18**	H-**Nlys**-NTrp-NTrp-**NLys**-Nspe-Nspe-**NLys**-Nspe-Nspe-**NLys**-NTrp-NTrp-NH_2	0.33	4	53.8
**19**	H-**NLys**-Nspe-Nspe-**NLys**-Nspe-Nspe-**NLys**-NTrp-NTrp-**NLys**-NTrp-NTrp-NH_2	0.33	4	52.8
**20**	H-**NLys**-Nspe-Nspe-**NLys**-NTrp-NTrp-**NLys**-NTrp-NTrp-**NLys**-Nspe-Nspe-NH_2	0.33	4	52.7
**21**	H-**NLys**-NTrp-NTrp-**NLys**-NTrp-NTrp-**NLys**-Nspe-Nspe-**NLys**-Nspe-Nspe-NH_2	0.33	4	53.2
**22**	H-**NLys**-Nspe-NTrp-**NLys**-Nspe-NTrp-**NLys**-Nspe-NTrp-**NLys**-Nspe-NTrp-NH_2	0.33	4	54.1
**23**	H-**Nlys**-NTrp-NTrp-**Nlys**-NTrp-NTrp-**Nlys**-NTrp-NTrp-**Nlys**-NTrp-NTrp-NH_2	0.33	8	53.0
**24**	H-**NLys**-Nspe-Nspe-**NLys**-Nspe-NTrp-**NLys**-Nspe-Nspe-**NLys**-**NLys**-Nspe-NH_2	0.42	1	49.4
**25**	H-**NLys**-Nspe-Nspe-**NLys**-Nspe-NTrp-**NLys**-**NLys**-Nspe-**NLys**-Nspe-Nspe-NH_2	0.42	1	50.8
**26**	H-**NLys**-Nspe-Nspe-**NLys**-**NLys**-NTrp-**NLys**-Nspe-Nspe-**NLys**-Nspe-Nspe-NH_2	0.42	1	49.5
**27**	H-**NLys**-**NLys**-Nspe-**NLys**-Nspe-NTrp-**NLys**-Nspe-Nspe-**NLys**-Nspe-Nspe-NH_2	0.42	1	50.7
**28**	H-**NLys**-Nspe-Nspe-**NLys**-Nspe-Nspe-**NLys**-Nspe-**NLys**-**NLys**-NTrp-NTrp-NH_2	0.42	2	48.6
**29**	H-**NLys**-Nspe-Nspe-**NLys**-Nspe-Nspe-**NLys**-**NLys**-Nspe-**NLys**-NTrp-NTrp-NH_2	0.42	2	48.6
**30**	H-**NLys**-Nspe-Nspe-**NLys**-**NLys**-Nspe-**NLys**-Nspe-Nspe-**NLys**-NTrp-NTrp-NH_2	0.42	2	48.8
**31**	H-**NLys**-**NLys**-Nspe-**NLys**-Nspe-Nspe-**NLys**-Nspe-Nspe-**NLys**-NTrp-NTrp-NH_2	0.42	2	50.2
**32**	H-**NLys**NTrp-NTrp-**NLys**-Nspe-Nspe-**NLys**-Nspe-Nspe-**NLys**-**NLys**-Nspe-NH_2	0.42	2	48.4
**33**	H-**NLys**-NTrp-NTrp-**NLys**-Nspe-Nspe-**NLys**-**NLys**-Nspe-**NLys**-Nspe-Nspe-NH_2	0.42	2	47.9
**34**	H-**NLys**-NTrp-NTrp-**NLys**-**NLys**-Nspe-**NLys**-Nspe-Nspe-**NLys**-Nspe-Nspe-NH_2	0.42	2	48.6
**35**	H-NTrp-NTrp-**NLys**-Nspe-Nspe-**NLys**-Nspe-Nspe-**NLys**-**NLys**-Nspe-**NLys**-Nspe-Nspe-NH_2	0.36	2	51.8
**36**	H-NTrp-NTrp-**NLys**-**NLys**-Nspe-**NLys**-Nspe-Nspe-**NLys**-Nspe-Nspe-**NLys**-Nspe-Nspe-NH_2	0.36	2	52.4
**37**	H-NTrp-NTrp-**NLys**-Nspe-Nspe-**NLys**-Nspe-Nspe-**NLys**-Nspe-Nspe-**NLys**-NH_2	0.33	2	52.7
**38**	H-NTrp-NTrp-**NLys**-Nspe-**NLys**-**NLys**-Nspe-Nspe-**NLys**-Nspe-Nspe-**NLys**-NH_2	0.42	2	47.8

[a] CTLR: charge-to-length-ratio. *N*-Terminal amine was not counted. [b] % MeCN.

Table S1 (continued). Sequences and properties of antimicrobial peptoids **39** − **66**

Comp'd	Sequence	CTLR[a]	# of *N*Trp	HPLC elution[b]
**39**	H-**NLys**-Nspe-Nspe-**NLys**-Nspe-Nspe-**NLys**-**NLys**-Npm-**NLys**-NTrp-NTrp-NH_2	0.42	2	47.8
**40**	H-**NLys**-Nspe-Nspe-**NLys**-Npm-Npm-**NLys**-**NLys**-Nspe-**NLys**-NTrp-NTrp-NH_2	0.42	2	47.5
**41**	H-**NLys**-Npm-Npm-**NLys**-Nspe-Nspe-**NLys**-**NLys**-Nspe-**NLys**-NTrp-NTrp-NH_2	0.42	2	47.5
**42**	H-Npm-**NLys**-Nspe-Nspe-**NLys**-Nspe-Nspe-**NLys**-Nspe-Nspe-**NLys**-Nspe-Nspe-Npm-NH_2	0.29	0	57.7
**43**	H-Npm-Npm-**NLys**-Nspe-Nspe-**NLys**-Nspe-Nspe-**NLys**-Nspe-Nspe-**NLys**-Nspe-Nspe-NH_2	0.29	0	56.8
**44**	H-**NLys**-Nspe-Nspe-**NLys**-Nspe-Nspe-**NLys**-Nspe-Nspe-**NLys**-Npm-Npm-NH_2	0.33	0	53.1
**45**	H-**NLys**-Nspe-Nspe-**NLys**-Nspe-Nspe-**NLys**-**NLys**-Nspe-**NLys**-Npm-Npm-NH_2	0.42	0	47.3
**46**	H-**NLys**-Npm-Npm-**NLys**-Nspe-Nspe-**NLys**-Nspe-Nspe-**NLys**-Nspe-Nspe-NH_2	0.33	0	53.7
**47**	H-**NLys**-Npm-Npm-**NLys**-Nspe-Nspe-**NLys**-Nspe-Nspe-**NLys**-**NLys**-Nspe-NH_2	0.42	0	47.8
**48**	H-Nspe-**NLys**-Nspe-Nspe-**NLys**-Nspe-Nspe-**NLys**-Nspe-Nspe-**NLys**-Nspe-NH_2	0.33	0	53.4
**49**	H-**NLys**-Nspe-Nspe-**NLys**-Nspe-Nspe-**NLys**-Nspe-Nspe-**NLys**-**NLys**-NH_2	0.45	0	46.6

**50**	H-**_N_Lys**-*N*spe-*N*spe-**_N_Lys**-*N*spe-*N*spe-**_N_Lys**-**_N_Lys**-*N*spe-**_N_Lys**-*N*spe-NH$_2$	0.45	0	46.1
**51**	H-**_N_Lys**-*N*spe-*N*spe-**_N_Lys**-**_N_Lys**-*N*spe-**_N_Lys**-*N*spe-*N*spe-**_N_Lys**-*N*spe-NH$_2$	0.45	0	45.3
**52**	H-**_N_Lys**-**_N_Lys**-*N*spe-**_N_Lys**-*N*spe-*N*spe-**_N_Lys**-*N*spe-*N*spe-**_N_Lys**-*N*spe-NH$_2$	0.45	0	46.9
**53**	H-*N*spe-**_N_Lys**-*N*spe-*N*spe-**_N_Lys**-*N*spe-*N*spe-**_N_Lys**-*N*spe-*N*spe-**_N_Lys**-**_N_Lys**-NH$_2$	0.42	0	48.5
**54**	H-*N*spe-**_N_Lys**-*N*spe-*N*spe-**_N_Lys**-*N*spe-*N*spe-**_N_Lys**-**_N_Lys**-*N*spe-**_N_Lys**-*N*spe-NH$_2$	0.42	0	48.1
**55**	H-*N*spe-**_N_Lys**-*N*spe-*N*spe-**_N_Lys**-**_N_Lys**-*N*spe-**_N_Lys**-*N*spe-*N*spe-**_N_Lys**-*N*spe-NH$_2$	0.42	0	47.2
**56**	H-*N*spe-**_N_Lys**-**_N_Lys**-*N*spe-**_N_Lys**-*N*spe-*N*spe-**_N_Lys**-*N*spe-*N*spe-**_N_Lys**-*N*spe-NH$_2$	0.42	0	48.0
**57**	H-*N*Trp-**_N_Lys**-*N*spe-*N*spe-**_N_Lys**-*N*spe-*N*spe-**_N_Lys**-*N*spe-*N*spe-**_N_Lys**-**_N_Lys**-NH$_2$	0.42	1	49.1
**58**	H-*N*Trp-**_N_Lys**-*N*spe-*N*spe-**_N_Lys**-*N*spe-*N*spe-**_N_Lys**-**_N_Lys**-*N*spe-**_N_Lys**-*N*spe-NH$_2$	0.42	1	48.7
**59**	H-*N*Trp-**_N_Lys**-*N*spe-*N*spe-**_N_Lys**-**_N_Lys**-*N*spe-**_N_Lys**-*N*spe-*N*spe-**_N_Lys**-*N*spe-NH$_2$	0.42	1	47.8
**60**	H-*N*Trp-**_N_Lys**-**_N_Lys**-*N*spe-**_N_Lys**-*N*spe-*N*spe-**_N_Lys**-*N*spe-*N*spe-**_N_Lys**-*N*spe-NH$_2$	0.42	1	48.6
**61**	H-*N*Trp-*N*spe-**_N_Lys**-*N*spe-*N*spe-**_N_Lys**-*N*spe-*N*spe-**_N_Lys**-*N*spe-*N*spe-**_N_Lys**-**_N_Lys**-NH$_2$	0.38	1	51.2
**62**	H-*N*Trp-*N*spe-**_N_Lys**-*N*spe-*N*spe-**_N_Lys**-*N*spe-*N*spe-**_N_Lys**-**_N_Lys**-*N*spe-**_N_Lys**-*N*spe-NH$_2$	0.38	1	50.8
**63**	H-*N*Trp-*N*spe-**_N_Lys**-*N*spe-*N*spe-**_N_Lys**-**_N_Lys**-*N*spe-**_N_Lys**-*N*spe-*N*spe-**_N_Lys**-*N*spe-NH$_2$	0.38	1	49.9
**64**	H-*N*Trp-*N*spe-**_N_Lys**-**_N_Lys**-*N*spe-**_N_Lys**-*N*spe-*N*spe-**_N_Lys**-*N*spe-*N*spe-**_N_Lys**-*N*spe-NH$_2$	0.38	1	50.5
**65**	H-**_N_4hb**-*N*Trp-*N*Trp-**_N_4hb**-*N*Trp-*N*Trp-**_N_4hb**-*N*Trp-*N*Trp-**_N_4hb**-*N*Trp-*N*Trp-NH$_2$	0	8	66.7
**66**	H-**_N_4hb**-*N*spe-*N*spe-**_N_4hb**-*N*spe-*N*spe-**_N_4hb**-**_N_4hb**-*N*spe-**_N_4hb**-*N*Trp-*N*Trp-NH$_2$	0	2	64.7
melittin	H-Gly-Ile-Gly-Ala-Val-Leu-Lys-Val-Leu-Thr-Thr-Gly-Leu-Pro-Ala-Leu-Ile-Ser-Trp-Ile-Lys-Arg-Lys-Arg-Gln-Gln-NH$_2$	-	-	55.4
buforin-II	H-Thr-Arg-Ser-Ser-Arg-Ala-Gly-Leu-Gln-Phe-Pro-Val-Gly-Arg-Val-His-Arg-Leu-Leu-Arg-Lys-NH$_2$	-	-	37.6
omiganan	H-Ile-Leu-Arg-Trp-Pro-Trp-Trp-Pro-Trp-Arg-Arg-Lys-NH$_2$	-	-	45.7
pexiganan	H-Gly-Ile-Gly-Lys-Phe-Leu-Lys-Lys-Ala-Lys-Lys-Phe-Gly-Lys-Ala-Phe-Val-Lys-Ile-Leu-Lys-Lys-NH$_2$	-	-	43.5

[a] CTLR: charge-to-length-ratio. *N*-Terminal amine was not counted. [b] % MeCN.

1
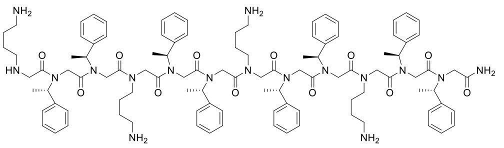

2
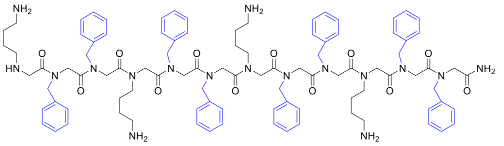

3
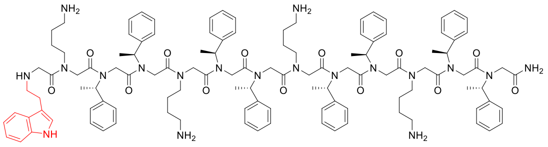

4
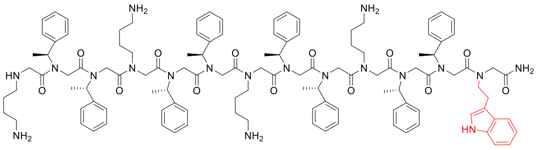

5
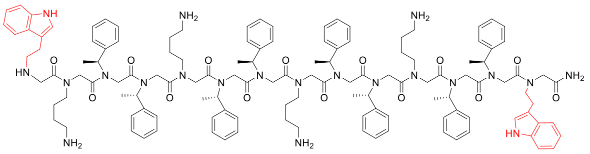

6
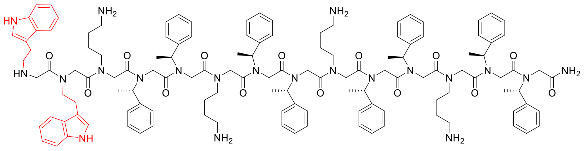

7
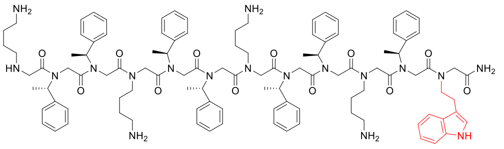

8
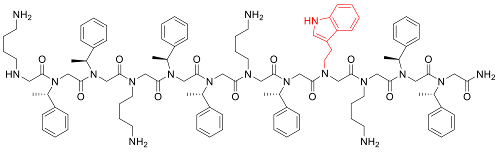

9
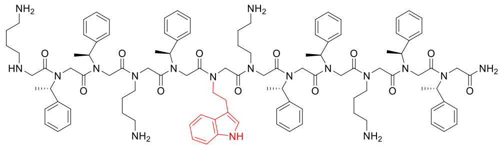

10
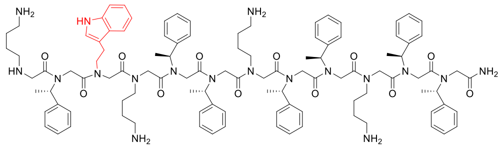

11
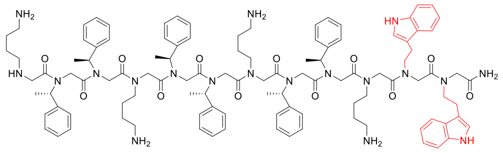

12
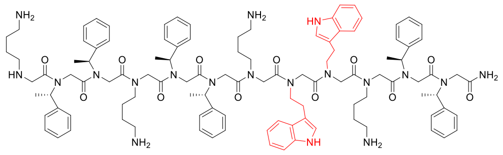

13
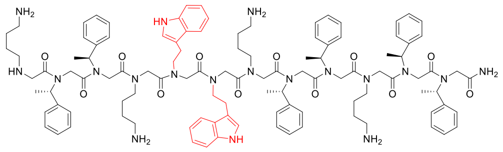

14
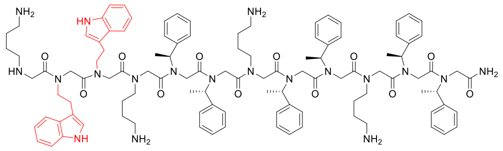

15
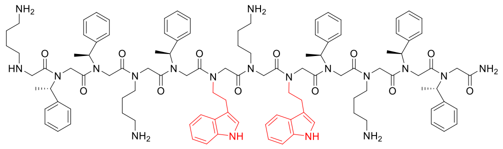

16
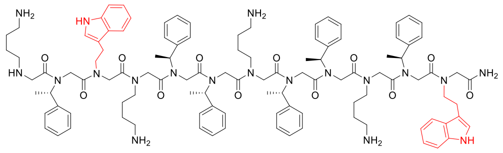

17

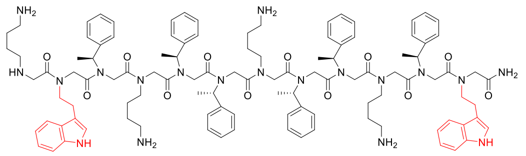

18

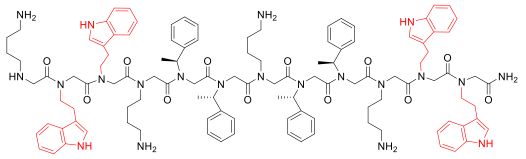

19

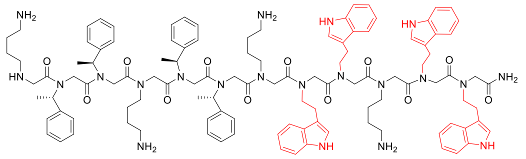

20

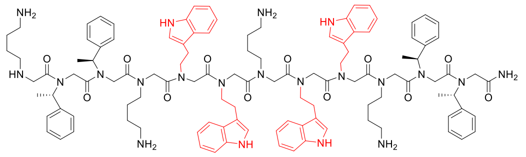

21

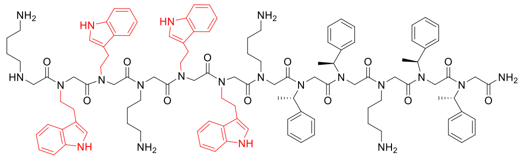

22

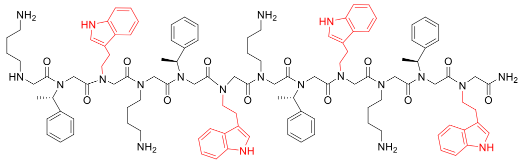

23

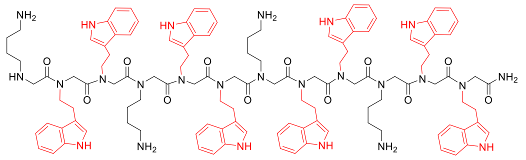

24

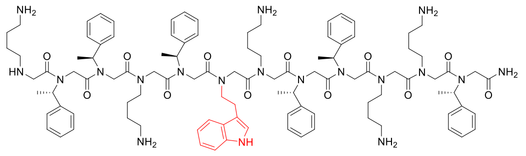

25

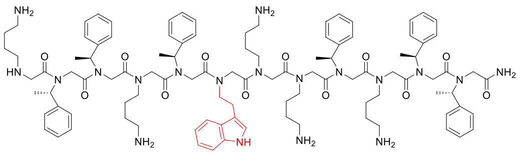

26

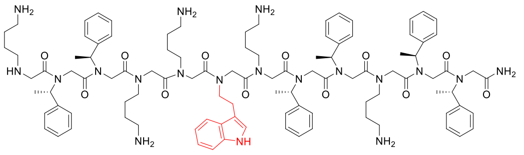

27

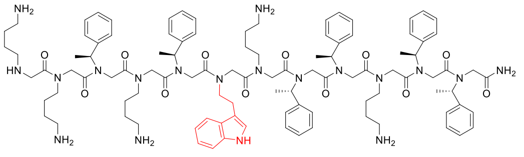

28

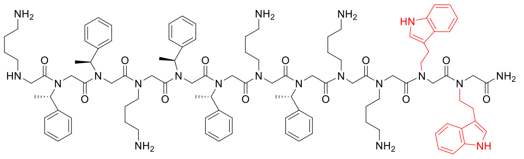

29

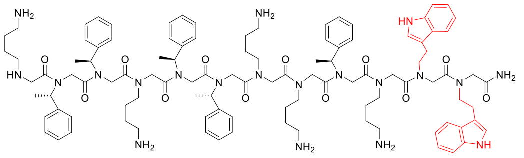

30

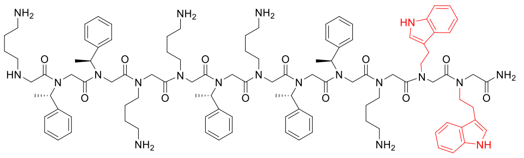

31

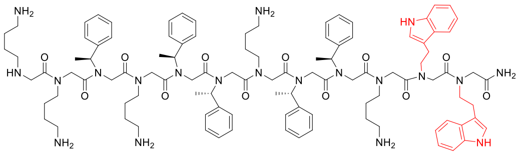

32

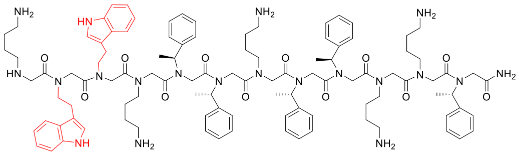

33

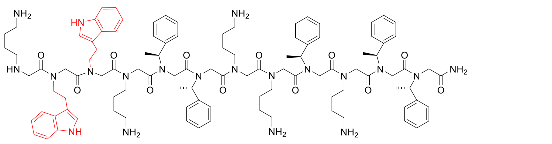

34

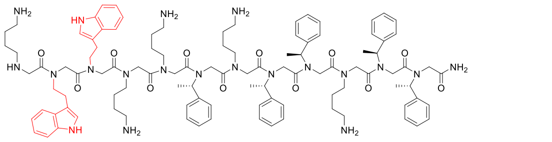

35

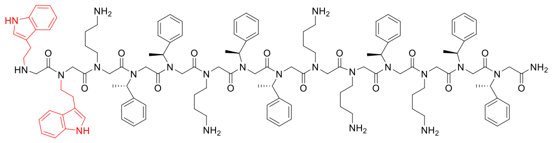

36

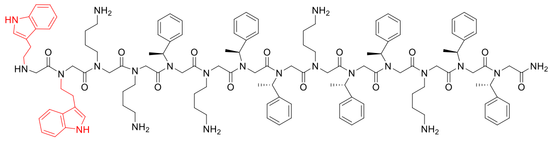

37

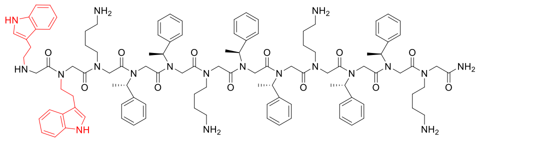

38

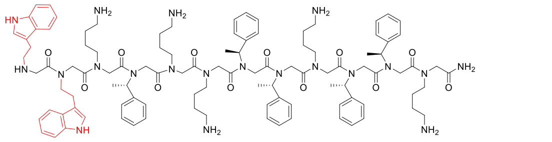

39

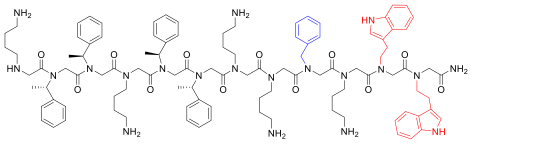

40

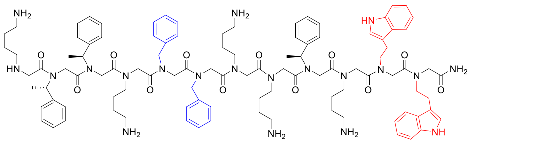

41

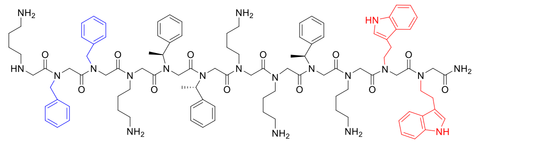

42

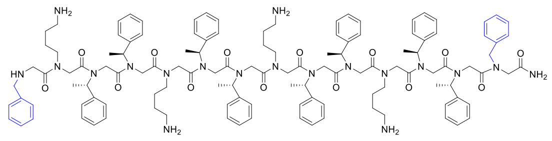

43

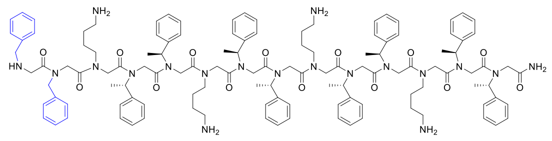

44

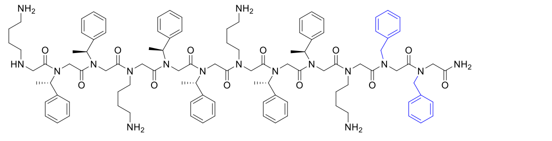

45

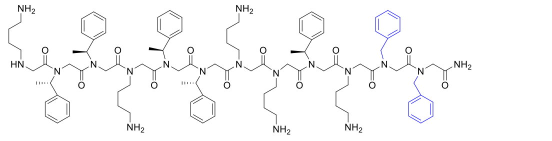

46

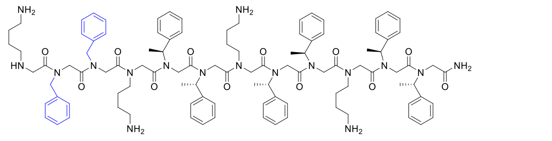

47

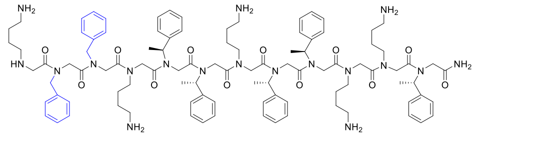

48

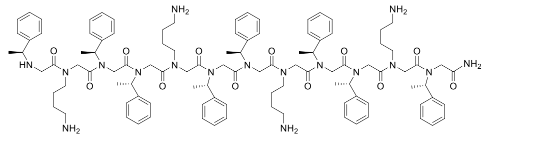

49 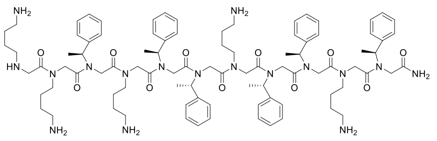

50 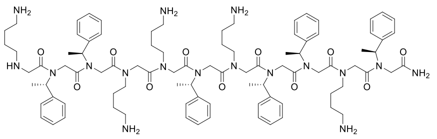

51 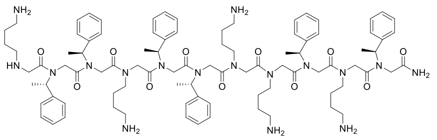

52 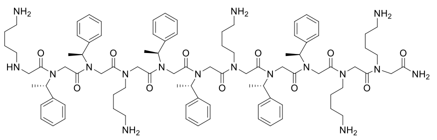

53 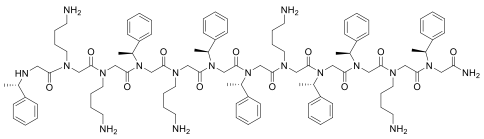

54 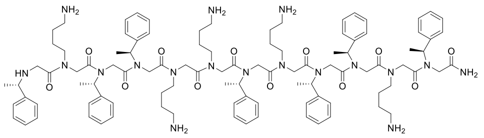

55 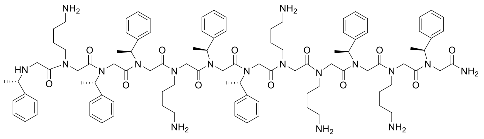

56 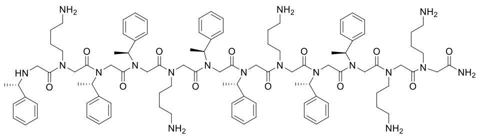

57 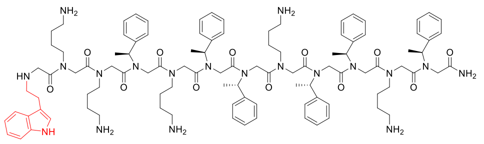

58 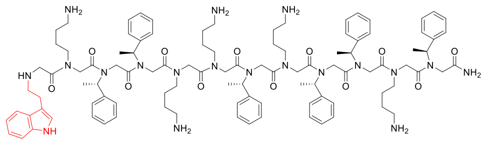

59 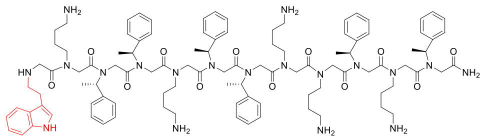

60 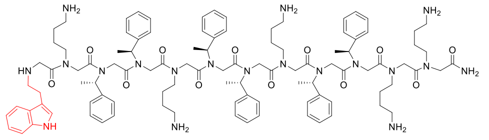

61 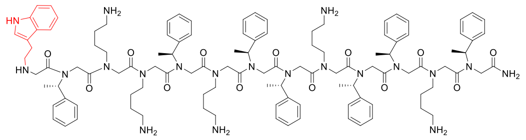

62 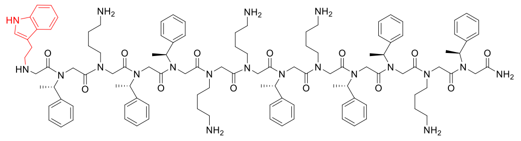

63 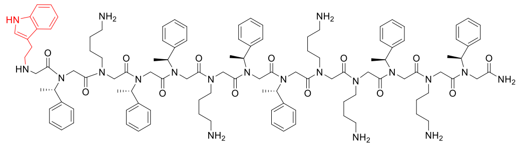

64 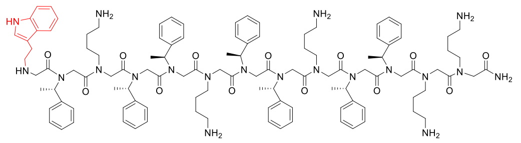

65 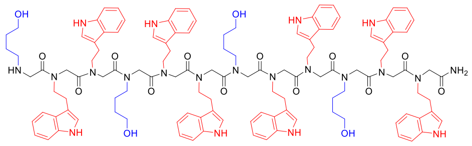

66 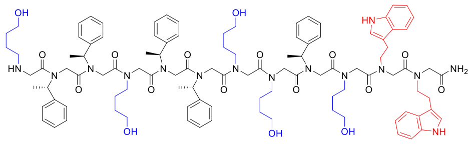

Figure S2. Chemical structures of peptoids **1**–**66**.

3. Antimicrobial and hemolytic activity

Table S2. Antimicrobial and hemolytic activities of **1 – 38**

Comp'd	MIC[a] (μM)		HC_{10} / HC_{50}[b] (μM)	H_{max}[c] (100 μM)	selectivity index[d]
	E. coli ATCC 25922	*S. aureus* ATCC 25923			
**1**	6.3	1.6	8.3/22.9	112.3±0.7	1.3
**2**	12.5	3.1	20.4/>100.0	17.7±1.1	1.6
**3**	6.3	1.6	6.3/13.9	100.5±9.9	1.0
**4**	12.5	1.6	<6.3/6.7	103.2±0.9	<0.5
**5**	>25	3.1	<6.3/15.3	108.1±2.7	<0.3
**6**	25	<0.8	9.6/22.0	101.6±5.8	0.4
**7**	6.3	3.1	7.8/23.8	94.1±4.3	1.2
**8**	6.3	1.6	12.8/28.4	105.1±2.2	2.0
**9**	3.1	1.6	10.6/31.9	106.4±0.6	3.4
**10**	3.1	1.6	6.6/17.0	108.1±1.7	2.1
**11**	6.3	1.6	8.3/22.2	103.4±3.2	1.3
**12**	6.3	1.6	29.1/65.2	87.4±2.4	4.6
**13**	6.3	<0.8	20.2/41.3	96.8±1.6	3.2
**14**	3.1	<0.8	23.6/43.4	90.0±7.3	7.6
**15**	6.3	1.6	28.1/60.9	91.0±2.5	4.5
**16**	6.3	1.6	13.8/25.0	100.3±1.7	2.2
**17**	6.3	1.6	18.0/39.7	87.3±6.7	2.9
**18**	12.5	3.1	14.3/37.8	103.5±4.3	1.1
**19**	12.5	0.8	3.8/12.5	101.5±1.3	0.3
**20**	6.3	1.6	8.2/33.9	106.7±3.5	1.3
**21**	6.3	1.6	23.2/64.9	83.7±2.0	3.7
**22**	12.5	1.6	6.9/19.4	108.6±1.1	0.6
**23**	25	1.6	17.1/45.2	72.2±0.3	0.7
**24**	>25	6.3	>100/>100	1.5±0.0	nd[e]
**25**	>25	3.1	>100/>100	2.7±0.4	nd[e]
**26**	>25	6.3	>100/>100	0.7±0.1	nd[e]
**27**	25	3.1	>100/>100	5.9±0.2	nd[e]
**28**	12.5	6.3	>100/>100	8.9±1.4	>8.0
**29**	6.3	6.3	>100/>100	9.8±1.8	>15.9
**30**	12.5	3.1	>100/>100	8.4±0.5	>8.0
**31**	6.3	3.1	59.0/>100	18.4±5.0	9.3
**32**	6.3	3.1	77.7/>100	15.9±1.5	12.3
**33**	12.5	3.1	>100/>100	5.0±0.2	>8.0
**34**	25	3.1	>100/>100	8.5±1.1	4.0
**35**	3.1	0.8	20.4/63.0	82.6±3.2	6.6
**36**	3.1	<0.8	15.3/39.4	89.1±3.4	4.9
**37**	3.1	1.6	21.1/57.0	97.4±6.5	6.8
**38**	12.5	6.3	>100/>100	5.9±0.5	>8.0

[a]These concentrations represent mean values in triplicate. [b]HC_{10} and HC_{50} are the concentrations of compounds causing 10% and 50% hemolysis in rat erythrocytes, respectively. These concentrations represent mean values in triplicate. [c]H_{max} is the percentage (%) of hemolysis at the highest concentration tested (100 μM). [d]The selectivity index was calculated as HC_{10} divided by the minimum inhibitory concentration (MIC) values in *E. coli* ATCC 25922. [e]Not determined.

Table S2 (continued). Antimicrobial and hemolytic activities of **39**–**66**

Comp'd	MIC[a] (μM)		HC_{10} / HC_{50}[b] (μM)	H_{max}[c] (100 μM)	selectivity index[d]
	E. coli ATCC 25922	*S. aureus* ATCC 25923			
**39**	25	6.3	100/>100	10.2±0.3	4.0
**40**	12.5	12.5	>100/>100	8.9±0.2	>8.0
**41**	25	6.3	>100/>100	7.1±0.1	>4.0
**42**	25	0.8	<3.1/6.6	105.9±1.1	<0.1
**43**	12.5	0.8	<3.1/6.4	105.6±3.8	<0.2
**44**	6.3	1.6	6.3/28.6	97.9±3.7	1.0
**45**	>25	25	>100/>100	4.5±0.2	nd[e]
**46**	6.3	0.8	6.9/36.5	90.8±2.7	1.1
**47**	25	12.5	>100/>100	4.8±0.2	>4.0
**48**	12.5	3.1	25.7/48.4	79.9±3.6	2.1
**49**	>25	>25	>100/>100	0.3±0.3	nd[e]
**50**	>25	>25	nd[e]	nd[e]	nd[e]
**51**	>25	>25	nd[e]	nd[e]	nd[e]
**52**	25	>25	nd[e]	nd[e]	nd[e]
**53**	25	>25	nd[e]	nd[e]	nd[e]
**54**	25	>25	nd[e]	nd[e]	nd[e]
**55**	25	>25	nd[e]	nd[e]	nd[e]
**56**	25	25	nd[e]	nd[e]	nd[e]
**57**	25	12.5	nd[e]	nd[e]	nd[e]
**58**	25	12.5	nd[e]	nd[e]	nd[e]
**59**	>25	25	nd[e]	nd[e]	nd[e]
**60**	>25	12.5	nd[e]	nd[e]	nd[e]
**61**	6.3	3.1	19.3/65.4	79.9±0.7	3.1
**62**	6.3	3.1	36.2/>100	44.8±8.1	5.7
**63**	12.5	3.1	85.0/>100	12.7±0.9	6.8
**64**	12.5	3.1	70.7/>100	18.9±2.9	5.7
**65**	>25	>25	nd[e]	nd[e]	nd[e]
**66**	>25	>25	nd[e]	nd[e]	nd[e]
melittin	12.5	≤1.6	<3.1/4.0	105.7±17.6	<0.2
buforin-II	12.5	12.5	nd[e]	nd[e]	nd[e]
omiganan	12.5	12.5	>100/>100	0	>8.0
pexiganan	3.1	≤1.6	>100/>100	8.2±0.6	>32.3

[a]These concentrations represent mean values in triplicate. [b]HC_{10} and HC_{50} are the concentrations of compounds causing 10% and 50% hemolysis in rat erythrocytes, respectively. These concentrations represent mean values in triplicate. [c]H_{max} is the percentage (%) of hemolysis at the highest concentration tested (100 μM). [d]The selectivity index was calculated as HC_{10} divided by the minimum inhibitory concentration (MIC) values in *E. coli* ATCC 25922. [e]Not determined.

4. Cell culture and cytotoxicity test using MTS assay

Human lung fibroblast cells (MRC-5) were purchased from the Korean Cell Line Bank (Seoul, South Korea), human keratinocytes (HaCaT) were provided by the Skin Research Institute R&D Center of Amore Pacific Company (Yongin, South Korea). All cells were grown in Dulbecco's modified Eagle’s medium (DMEM) supplemented with 10% fetal bovine serum at 37 °C in an atmosphere containing 5% CO_2. The 3-(4,5-dimethylthiazol-2-yl)-5-(3-carboxymethoxyphenyl)-2-(4-sulfophenyl)-2H-tetrazolium (MTS) cell viability assays were used to determine the effects of peptoids on cell growth.[4] Aliquots (100 µL) of media containing 1.3×10^4 MRC-5 cells/mL or 3×10^4 HaCaT cells/mL were distributed into each well of a 96-well plate (Eppendorf, Hamburg, Germany). The cells were allowed to attach and were grown in a humidified atmosphere containing 5% CO_2 at 37 °C for 24 h. When the cell density reached approximately 70% confluency, the medium was replaced with serial dilutions of the peptoid stock in DMEM and incubated for an additional 24 h. At this point, 20 µL of the CellTiter 96 aqueous non-radioactive cell proliferation assay reagent (Promega, Madison, WI, USA), which contains the tetrazolium compound, was added to each well. Plates were incubated for 4 h at 37 °C to allow time for metabolism. The MTS-formazan products in viable cells were measured at 490 nm using a microplate reader, and the percentage of cell viability (%) was calculated by comparing readings to the values of untreated wells. Percentage values of cell viability were calculated as A/(A control)×100, where A is the absorbance of the test well and A control is the average absorbance of wells containing untreated cells.

5. Structure-activity relationship (SAR) analysis

Peptoids **1** and **2** with known α-helical or non-helical structures, respectively, were used as references.[5] Using the helical peptoid **1** as a lead sequence, *N*Trp was either added (**3**–**6**) or substituted (**7**–**23**). The number (1, 2, 4, and 8 *N*Trps) and positions (*N*- or *C*-termini or central) of the added or substituted *N*Trp varied to determine the correlation between *N*Trp and antimicrobial activity. In peptoids **3** and **4**, one *N*Trp was added to the *N*-terminus or *C*-terminus, respectively. In **5** and **6**, two *N*Trp’s were added to both termini or to the *N*-terminus. In peptoids **7** to **10**, a single *N*Trp substituted an *N*spe in peptoid **1** in a sequential order from the *C*- to *N*-terminus. Four peptoids, from **11** to **14**, contained two adjacent *N*Trp residues by substituting two *N*spe’s in the same sequence of substitutions. In **15**–**17**, two non-

adjacent *N*spe's were substituted by two *N*Trp's. As the compound number increases, the distance between the two *N*Trp's becomes greater. Five peptoids, **18**–**22**, contained four *N*Trp's. Peptoid **22** had four repeats of *N*Lys-*N*spe-*N*Trp unit and did not contain adjacent *N*Trp residues. Finally, all *N*spe's were substituted with eight *N*Trp residues in peptoid **23**.

When the *N*Trp residue was added at the *N*- or *C*-terminus (**3**–**6**) of peptoid **1**, MIC values for *E. coli* generally increased (except for **3**), but these peptoids showed decreased selectivity due to severe hemolysis. Peptoids **9** and **10**, with one *N*Trp substitution, exhibited increased antimicrobial activity (MIC = 3.1 μM) with a marginal increase in selectivity over peptoid **1**. Interestingly, peptoid **14**, containing two adjacent *N*Trp's (WW motif) as an *N*-terminal substitution, showed increased antimicrobial activity and selectivity, but peptoid **11** containing two *N*Trp's at the *C*-terminus had a very similar MIC and selectivity index as peptoid **1**. Peptoids containing two separate *N*Trp's without a WW motif (**15**–**17**) exhibited the same MIC values as peptoid **1** with slight gains in selectivity. Substitutions containing more than two *N*Trp residues (**18**–**23**) did not show any increase in activity or selectivity.

Peptoids **3**–**23** contained four *N*Lys residues (i.e., fixed cationic charges) and showed HPLC elution at 53~58% MeCN indicating increased hydrophobic character of these peptoids. This resulted in strong hemolytic activity and poor selectivity. The MIC of the 12mer peptoids **7**–**23** (with the same CTLR value of 0.33) was analyzed to determine the effect of the number of *N*Trp monomer(s) on the antibacterial action against *E. coli* (Figure S3). This series of experiments showed that peptoids containing one or two *N*Trp monomer(s) exhibited more potent antimicrobial activity against *E. coli.*

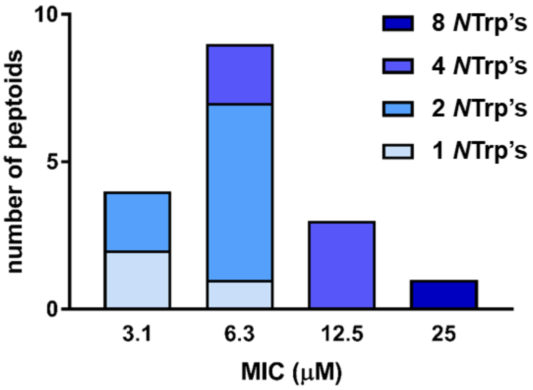

Figure S3. Effect of the number of *N*Trp monomer(s) on the *E. coli* MIC value. Peptoids **7** – **23** were analyzed, representing sequence variants of peptoid **1** with the same length (CTLR = 0.33).

Previously, we observed that an increased cationic charge in antimicrobial peptoids generally led to selective interaction with the negatively charged bacterial membrane and simultaneously reduced toxicity against eukaryotic cells.[6] Peptoids **24**–**27** had one additional *N*Lys due to the sequential replacement of an *N*spe in peptoid **9**. While these substituted compounds maintained antimicrobial activity against *S. aureus*, their antimicrobial activity against *E. coli* decreased. At the same time these compounds showed reduces hemolysis. Peptoids **11** and **14** exhibited potent antimicrobial activity. The cationic charge of these compounds was increased by replacing one *N*spe with an *N*Lys residue, providing peptoids **28**–**31** and **32**–**34**, respectively. These seven peptoids had an increased cationic charge and contained a WW motif. Among these, peptoids **29** and **32** retained antimicrobial activity while significantly increasing selectivity (i.e., selectivity index of >15.9 for **29** and 12.3 for **32**). It should be noted that these sequences with the same monomer compositions but with varying positions of the WW-motif and *N*Lys resulted in very different activities (e.g., MIC (*E. coli*) of **34** and hemolytic activity of **31**).

In peptoids **35** and **36**, one *N*spe of **6** was substituted with *N*Lys, providing significantly improved activity against *E. coli* and *S. aureus*, but the selectivity index still remained in the single digit range. For peptoids **37** and **38**, the sequences in **11** and **29** were reversed, but overall activity and selectivity did not improve over **29**. The helicity of **29** was modulated by substituting chiral *N*spe with achiral *N*pm monomer, resulting in peptoids **39**–**41**. The moderate helicity provided less toxicity, reduced hemolysis, but the helicity modulation also resulted in decreased antimicrobial activity, leading to suboptimal selectivity (i.e., selectivity index lower than 10).

Notably, the peptoids in this library were mostly active against gram-positive *S. aureus* with MIC values in the range of 0.8 – 3.1 μM. However, we initially focused on the activity against gram-negative *E. coli* and selectivity (i.e., lack of hemolytic activity) to select the best peptoid. These considerations resulted in the selection of peptoids **29** and **32** as hit compounds in this library screening.

To elucidate the role of the indole side chain, peptoids **42**–**47** were synthesized as controls of peptoids **5**, **6**, **11**, **29**, **14**, and **32**, respectively, where *N*Trp(s) was (or were) substituted with *N*pm(s). Interestingly, the two best peptoids in the library, **29** and **32**, lost the antimicrobial activity when the WW motif was replaced by two phenyls (two *N*pm's) as demonstrated by **45** and **47**, respectively. This result highlights the importance of two adjacent *N*Trp residues (WW motif) in the antimicrobial activity of **29** and **32**.

In case of peptoids **48-64**, further variations in lengths, positions, and the number of *N*Lys or *N*Trp monomers were made. Finally, in peptoids **65** and **66**, the *N*Lys residues of **23** and **29** were substituted by the hydroxyl containing *N*4hb residues, resulting in the complete loss of antimicrobial activity.

6. Effect of counter-ion exchange on antimicrobial, hemolytic, and cytotoxic activity

Counter-ion exchange procedure

For the counter-ion exchange, trifluoroacetate (TFA) ions were removed using a carbonate ion-exchange resin (VariPure columns, Agilent, Santa Clara, CA, USA) according to the manufacturer's instruction and following previously reported procedures.[7]

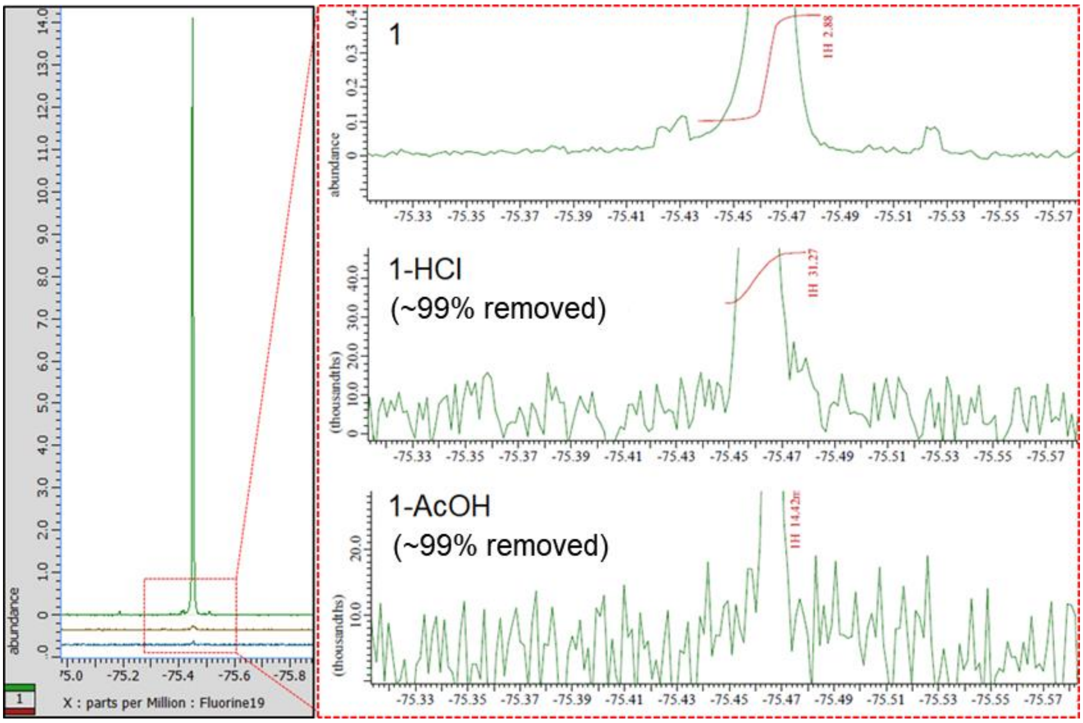

Figure S4. Quantitative analysis of ^{19}F-NMR after counter-ion exchange

The effectiveness of counter-ion exchange was verified using ^{19}F-NMR spectroscopy by measuring the amount of fluorine in the residual TFA (Figure S4). Quantitative NMR analysis was performed for peptoid **1** (**1** (as a TFA salt), **1**-HCl, and **1**-AcOH), and the amount of fluorine in **1**-HCl and **1**-AcOH was quantified by comparing the peak area of ^{19}F of TFA. In the prepared NMR solution, the fluorine molarity ratios were 2.88, 0.031, and 0.014 in **1** (TFA), **1**-HCl, and **1**-AcOH, respectively. These tests confirmed that ~99% of

trifluoroacetate was exchanged with chloride in **1**-HCl and ~99% of trifluoroacetate was exchanged with acetate in **1**-AcOH.

After counter-ion exchange, HPLC chromatograms were analyzed to confirm the purity of the peptoids (Figure S5). HPLC data indicated that exchange with acetate occurred without any degradation (>99% purity). However, a minor degradation was observed during counter-ion exchange with HCl (>95% purity).

The antimicrobial, hemolytic, and cytotoxic activities of counter-ion-exchanged peptoids were evaluated (Table S3). The results indicated that there were differences between the three different salts, although these changes did not conform to a consistent pattern. Generally, counter-ion-exchanged peptoids maintained their antimicrobial activity, although peptoids **10**, **15**, and **37** exhibited decreased antimicrobial activity after exchanging TFA to HCl or AcOH. There was no general trend regarding the hemolytic activity and eukaryotic cytotoxicity depending on the final salt form of peptoids. Therefore, we suggest that individual salt forms have to be examined for *in vitro* efficacy and toxicity before selecting a potential lead compound.

The impact of TFA was investigated in animal models previously. Pini et al. compared the effect of counter-ions (TFA and AcOH) on the antimicrobial efficacy and toxicity of theantimicrobial peptide M33 (KKIRVRLSA).[8] In terms of efficacy, TFA and AcOH salts did not differ substantially *in vitro* and *in vivo*. However, M33-TFA was 5-30% more toxic than M33-AcOH in human cells and in animals. In our hands, significantly reduced hemolytic activity was observed with **29**-AcOH compared to **29** (TFA), without any change in antibacterial effectiveness *in vitro*. Interestingly, this result confirms Pini’s findings, where counter-ion exchanging from TFA to AcOH did not affect efficacy but improved toxicity of the peptide they were testing.

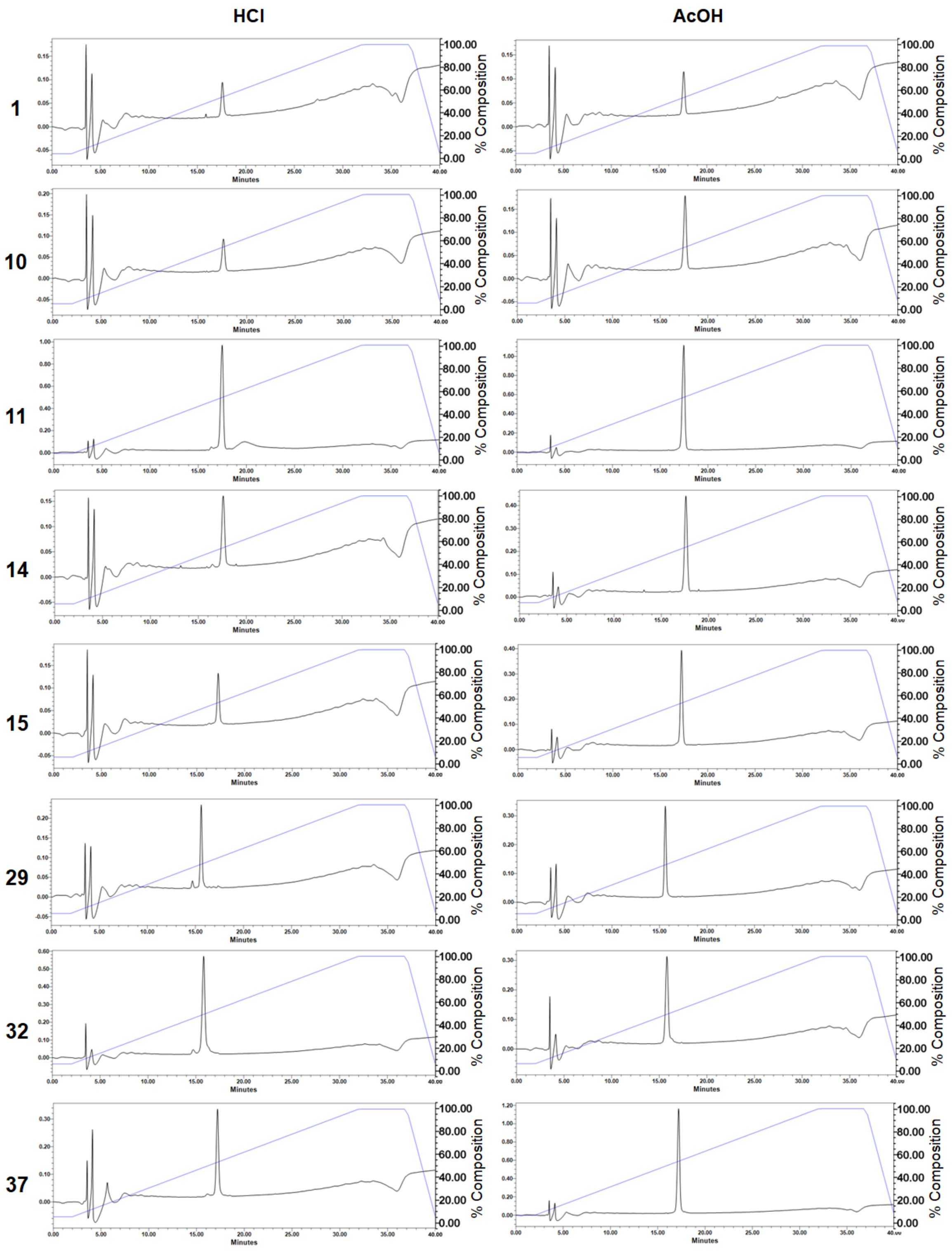

Figure S5. HPLC chromatograms of counter-ion exchanged peptoids with UV detection at 220 nm

Table S3. Antimicrobial, hemolytic, and cytotoxic activities of counter-ion-exchanged peptoids

Comp'd	MIC[a] (μM)		HC_{10} / HC_{50}[b] (μM)	H_{max}[c] (100 μM)	selectivity index[d]	LC_{50}[e] (μM)	
	E. coli ATCC25922	*S. aureus* ATCC25923				MRC-5	HaCaT
**1**	6.3	1.6	15.9/49.0	72.1±2.1	2.5	8.1	7.3
**1-HCl**	6.3	1.6	27.6/83.3	61.5±0.9	4.4	11.8	nd[f]
**1-AcOH**	12.5	3.1	25.0/78.6	58.2±1.7	2.0	8.6	nd[f]
**10**	3.1	0.8	6.9/25.8	101.4±5.1	2.2	nd[f]	nd[f]
**10-HCl**	6.3	3.1	15.3/37.8	81.5±7.4	2.4	nd[f]	nd[f]
**10-AcOH**	6.3	1.6	14.1/29.4	102.3±11.3	2.2	nd[f]	nd[f]
**11**	3.1	1.6	6.9/27.5	111.9±2.7	2.2	6.9	nd[f]
**11-HCl**	3.1	3.1	10.1/21.5	113.9±1.7	3.3	9.6	nd[f]
**11-AcOH**	3.1	1.6	8.7/29.7	110.4±0.2	2.8	8.3	nd[f]
**14**	3.1	1.6	12.5/21.1	112.9±2.1	4.0	9.7	nd[f]
**14-HCl**	3.1	3.1	19.2/68.2	75.3±5.0	6.2	12.1	nd[f]
**14-AcOH**	3.1	1.6	14.9/51.8	100.7±17.2	4.8	13.8	nd[f]
**15**	3.1	1.6	20.5/89.2	53.4±3.1	6.6	nd[f]	nd[f]
**15-HCl**	6.3	3.1	50.4/>100	33.4±9.7	8.0	nd[f]	nd[f]
**15-AcOH**	6.3	3.1	39.2/>100	34.1±1.1	6.2	nd[f]	nd[f]
**29**	6.3	12.5	88.6/>100	12.3±1.3	14.1	11.8	18.8
**29-HCl**	12.5	12.5	>100/>100	4.3±0.3	>8.0	23.9	>25.0
**29-AcOH**	6.3	12.5	>100/>100	5.2±0.2	>15.9	16.0	22.0
**32**	6.3	3.1	>100/>100	4.2±1.0	>15.9	14.5	17.1
**32-HCl**	6.3	6.3	>100/>100	8.7±0.6	>15.9	18.1	21.4
**32-AcOH**	6.3	6.3	83.0/>100	13.8±1.1	13.2	16.9	21.3
**37**	3.1	1.6	13.2/38.6	80.7±12.8	4.3	nd[f]	nd[f]
**37-HCl**	6.3	3.1	14.4/56.5	86.6±5.7	2.3	nd[f]	nd[f]
**37-AcOH**	6.3	1.6	12.5/26.0	98.9±8.0	2.0	nd[f]	nd[f]

[a]These concentrations represent mean values in triplicate. [b]HC_{10} and HC_{50} are the concentrations of compounds causing 10% and 50% hemolysis in rat erythrocytes, respectively. These concentrations represent mean values in triplicate. [c]H_{max} is the percentage (%) of hemolysis at the highest concentration tested (100 μM). [d]The selectivity index was calculated as HC_{10} divided by the minimum inhibitory concentration (MIC) values in *E. coli* ATCC 25922. [e]LC_{50} are the concentrations of compounds causing 50% lethality in the cells. [f]Not determined.

7. HPLC chromatogram, retention time (t_R), and LC-MS data

HPLC characterization

HPLC analysis of peptoids was conducted using a Waters HPLC system equipped with a Waters 2489 UV/Visible Detector, 1525 Binary HPLC Pump, 2707 Autosampler, 5CH column oven, and a C18 column (SunFire C18, 4.6 × 250 mm, 5 μm; Waters Corp., Milford, MA, USA). The mobile phases were deionized water (A, with + 0.1% TFA) and acetonitrile (ACN; B, with + 0.1% TFA). Before sample injection, the column was conditioned with 5% B for 10 min. Then, the mobile phase was maintained at 5% B for 2 min, followed by increasing B to 100% using a linear gradient over 30 min. At this point, 100% B was maintained for 5 additional min. The flow rate of the mobile phase was 1 mL/min. The sample was detected by measuring absorbance at 220 nm. The chromatograms of the peptoids used in this study are presented in Figure S5.

Crude peptoids were purified using a preparative HPLC system (Waters 2545 Quaternary Gradient Module, 2489 UV/Visible Detector, fraction collector III) with a C18 column (SunFire C18, 19 × 150 mm, 5 μm). Purification was conducted at a flow rate of 14 mL/min. The absorbance was measured at 220 and 254 nm to monitor sample elution. Analytical HPLC confirmed the purity of the peptoid products in each fraction. Fractions containing the pure product (>96 % purity) were collected, lyophilized and stored at -80 °C.

Reversed-phase HPLC chromatograms of purified peptoids and their retention times are shown in Figure S6. Changes in HPLC retention times allowed the approximate comparison of the hydrophobicity of peptoids (Table S1). Starting from peptoid **1**, the addition of a *N*Trp residue resulted in increased retention time (**3**–**6**). Substitution of *N*spe with *N*Trp did not result in a definite trend in retention time change. As expected, additional cationic charge by the incorporation of *N*Lys residue or the substitution of *N*spe with *N*Lys always led to a decreased retention time. For example, peptoid **29** (t_R = 48.6 min) had decreased retention time compared to peptoid **11** (t_R = 54.0 min). Notably, substitution of *N*Lys amine groups with *N*4hb hydroxyl groups resulted in a significant increase in retention time as shown in peptoid **63** (t_R = 66.7 min) and **64** (t_R = 64.7 min) (Table S1), which leads to poor aqueous solubility of the two peptoids.

The retention time of peptoid **1** (t_R = 54.5 min) was similar to that of melittin (t_R = 55.4 min), interestingly both causing severe membrane lysis in eukaryotic cells. The retention time of peptoid **29** was comparable to that of omiganan (t_R = 45.7 min) and both of these were relatively selective.

The identity of the synthesized peptoids was confirmed by electrospray ionization-mass spectrometry (Table S4).

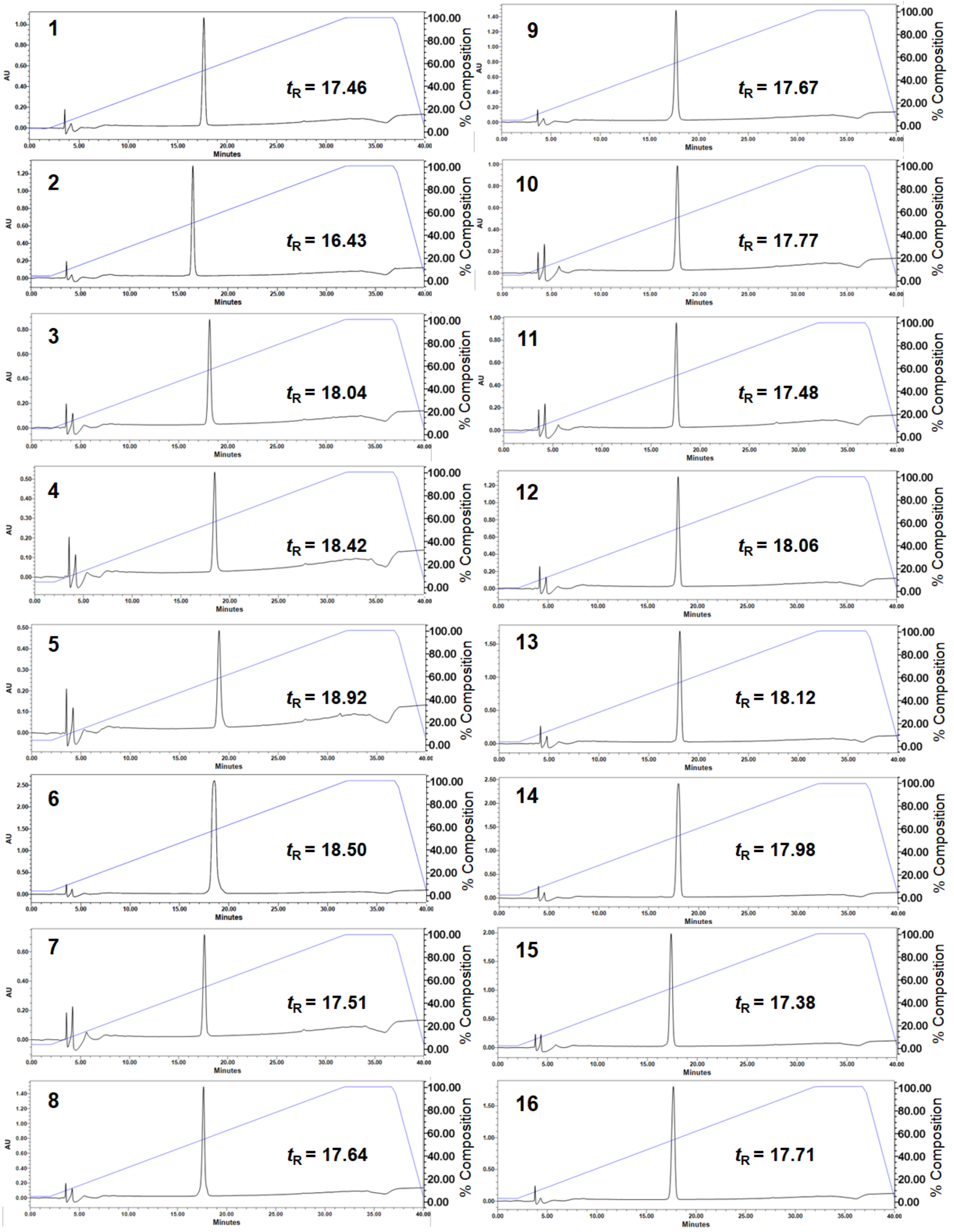

Figure S6. HPLC chromatograms of peptoids **1 - 66** with UV detection at 220 nm

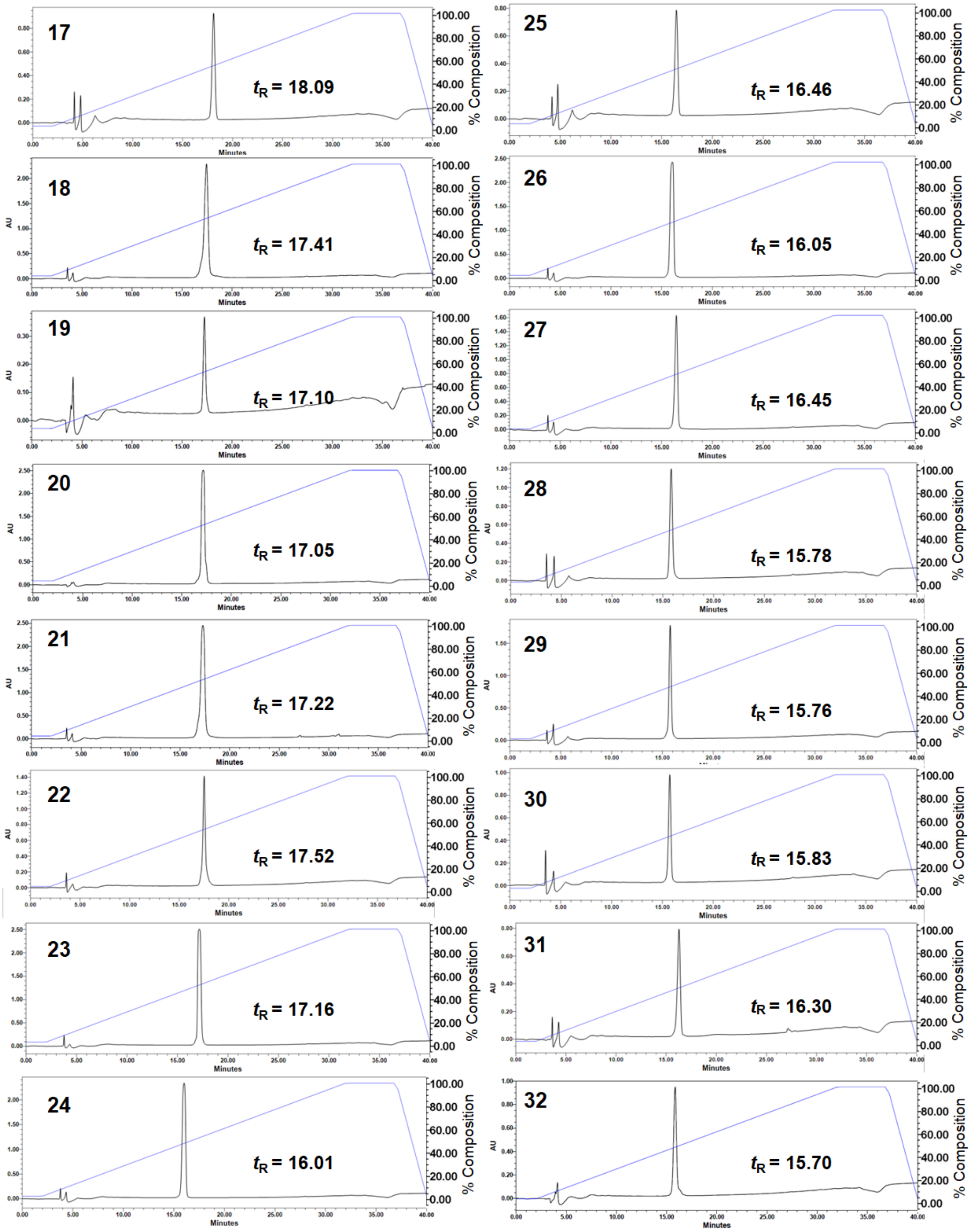

Figure S6 (continued)**.** HPLC chromatograms of peptoids **1 - 66** with UV detection at 220 nm

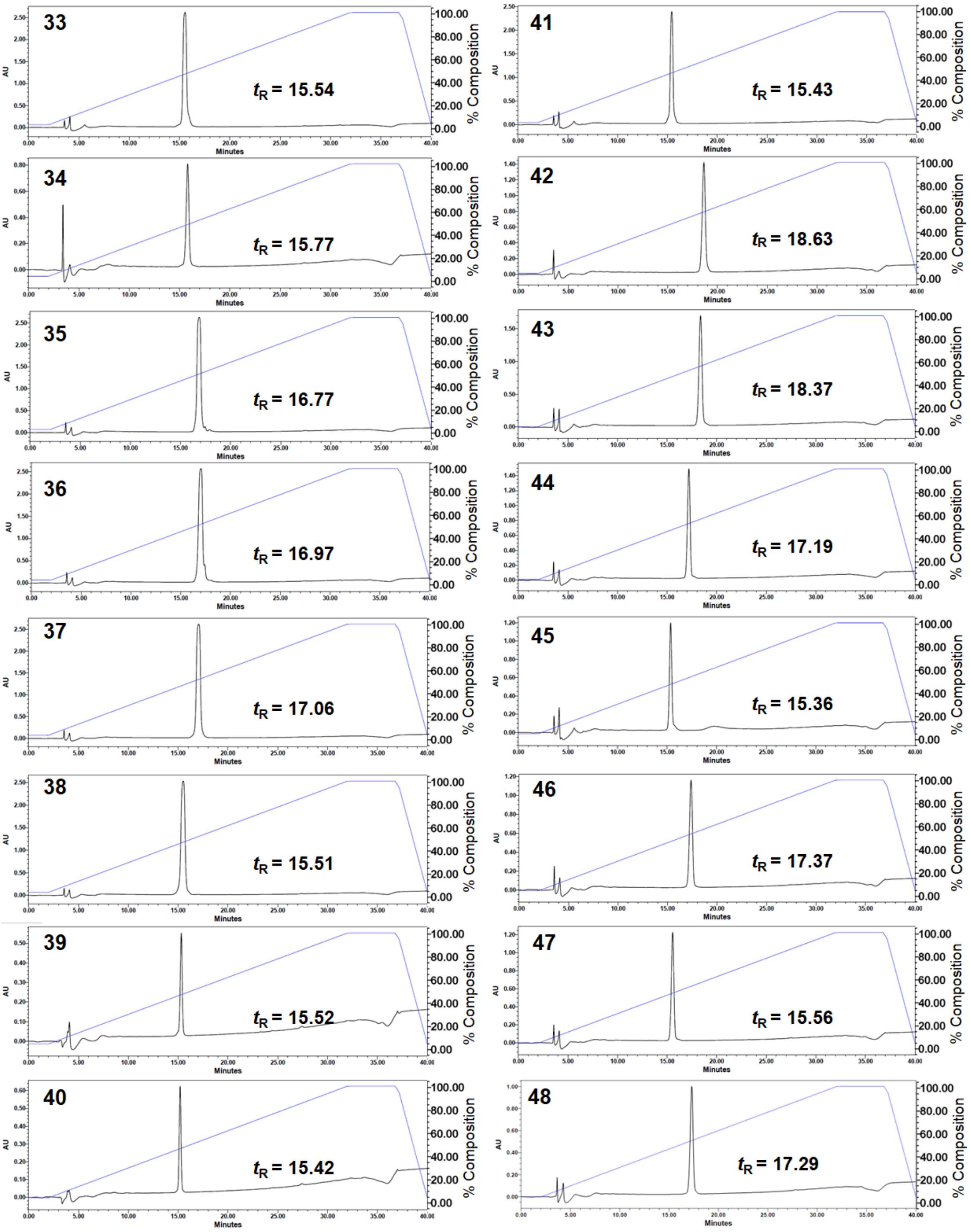

Figure S6 (continued)**.** HPLC chromatograms of peptoids **1 - 66** with UV detection at 220 nm

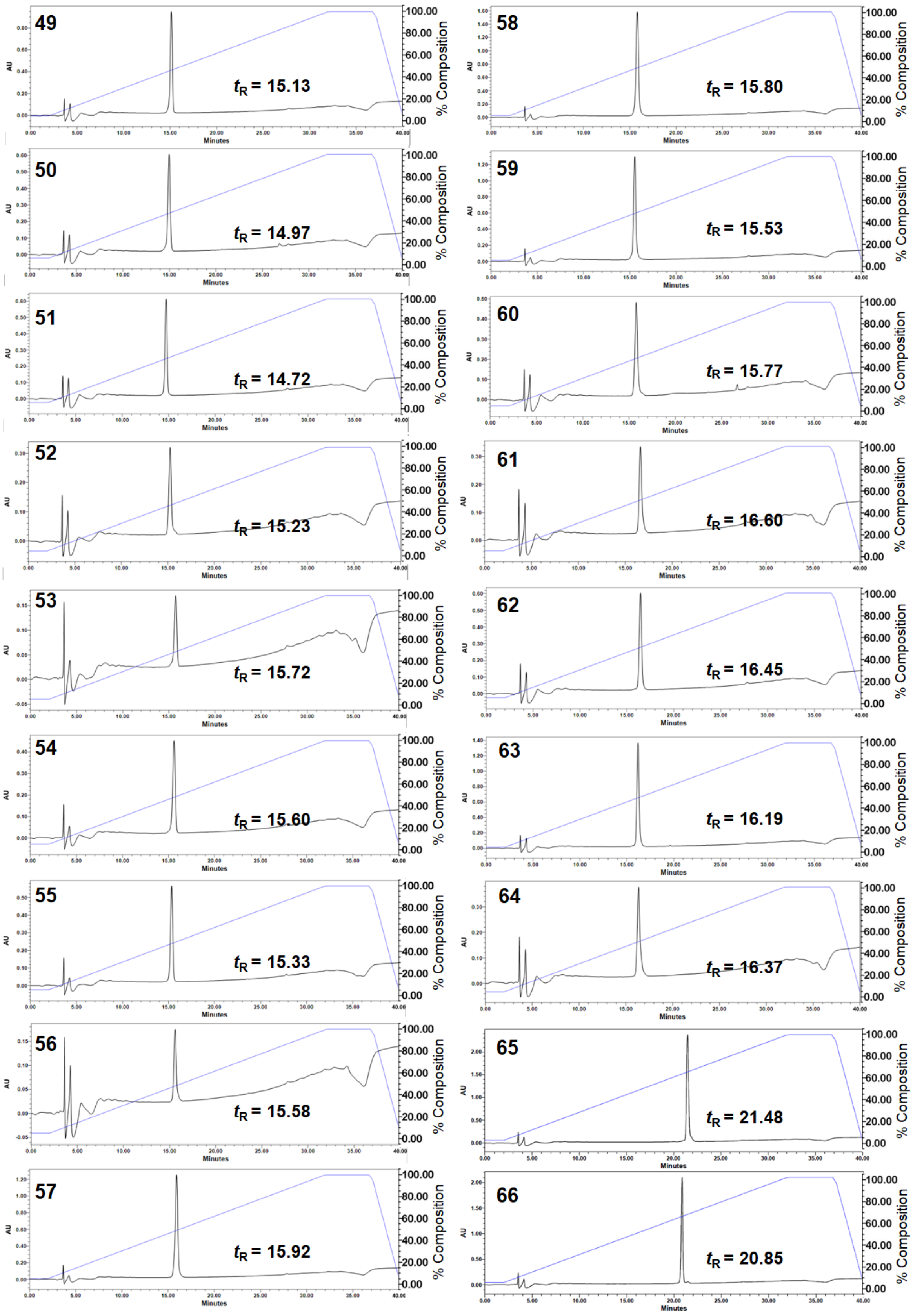

Figure S6 (continued)**.** HPLC chromatograms of peptoids **1 - 66** with UV detection at 220 nm

LC-MS characterization

An Agilent LC-MS system (1260 Infinity LC and 6120 Quadrupole MS with API-electrospray ion source, Agilent, Santa Clara, CA, USA) equipped with C18 column (Agilent C18, 4.6 × 50 mm, 2.7 μm) operated at 25 °C was used to obtain the mass data of peptoids via gradual increments in ACN composition using a binary mobile phase system (A: deionized water + 0.1% TFA, B: ACN + 0.1% TFA).

Table S4. Electrospray ionization-mass spectrometry data of peptoids **1 - 66**

Compound number	Calculated mass		Observed mass	
	$[M+H]^+$	$[M+2H]^{2+}$	$[M+H]^+$	$[M+2H]^{2+}$
**1**	1819.1	910.1	1819.9	910.5
**2**	1707.0	854.0	1707.8	854.2
**3**	2019.2	1010.1	*nd	1010.2
**4**	2019.2	1010.1	*nd	1010.8
**5**	2219.3	1110.2	*nd	1110.8
**6**	2219.3	1110.2	*nd	1110.7
**7**	1858.1	929.6	1858.9	930.0
**8**	1858.1	929.6	1858.9	930.0
**9**	1858.1	929.6	1858.9	929.6
**10**	1858.1	929.6	1858.9	930.0
**11**	1897.1	949.1	1897.9	949.1
**12**	1897.1	949.1	1898.0	949.2
**13**	1897.1	949.1	1898.0	949.2
**14**	1897.1	949.1	1897.9	949.2
**15**	1897.1	949.1	1898.0	949.6
**16**	1897.1	949.1	1898.0	949.3
**17**	1897.1	949.1	1897.9	949.2
**18**	1975.1	988.1	1976.0	988.5
**19**	1975.1	988.1	1976.0	988.5
**20**	1975.1	988.1	1976.0	988.6
**21**	1975.1	988.1	1976.0	988.7

**22**	1975.1	988.1	1976.0	988.2
**23**	2131.2	1066.1	*nd	1066.6
**24**	1825.1	913.1	1826.0	913.2
**25**	1825.1	913.1	1825.9	913.6
**26**	1825.1	913.1	1825.9	913.2
**27**	1825.1	913.1	1826.0	913.2
**28**	1864.1	932.6	1864.9	932.6
**29**	1864.1	932.6	1865.0	932.7
**30**	1864.1	932.6	1865.0	933.0
**31**	1864.1	932.6	1865.0	933.1
**32**	1864.1	932.6	1864.9	933.0
**33**	1864.1	932.6	1864.9	932.7
**34**	1864.1	932.6	1865.0	932.7
**35**	2186.3	1093.7	*nd	1094.2
**36**	2186.3	1093.7	*nd	1094.2
**37**	1897.1	949.1	1897.9	949.2
**38**	1864.1	932.6	1864.9	933.1
**39**	1850.1	925.6	1851.0	925.7
**40**	1836.1	918.6	1836.9	919.0
**41**	1836.1	918.6	1836.9	919.1
**42**	2113.2	1057.1	*nd	1057.3
**43**	2113.2	1057.1	*nd	1057.3
**44**	1791.1	896.1	1791.9	896.6
**45**	1758.1	879.6	1758.9	879.7
**46**	1791.1	896.1	1791.9	896.6
**47**	1758.1	879.6	1758.9	880.1
**48**	1819.1	910.1	1818.9	910.1
**49**	1625.0	813.0	1624.8	813.1
**50**	1625.0	813.0	1625.8	813.1
**51**	1625.0	813.0	1625.8	813.2
**52**	1625.0	813.0	1625.8	813.5
**53**	1786.1	893.6	1786.9	893.6
**54**	1786.1	893.6	1786.9	893.6
**55**	1786.1	893.6	1786.9	893.6

**56**	1786.1	893.6	1786.9	893.6
**57**	1825.1	913.1	1826.0	913.2
**58**	1825.1	913.1	1825.9	913.2
**59**	1825.1	913.1	1825.9	913.6
**60**	1825.1	913.1	1825.9	913.2
**61**	1986.2	993.6	1987.0	993.7
**62**	1986.2	993.6	1987.0	993.8
**63**	1986.2	993.6	1987.0	993.7
**64**	1986.2	993.6	1987.0	994.0
**65**	2135.1	1068.1	*nd	1068.3
**66**	1869.0	935.0	*nd	935.4

*nd: singly charged mass was not detected.

8. Growth kinetics against *P. aeruginosa*

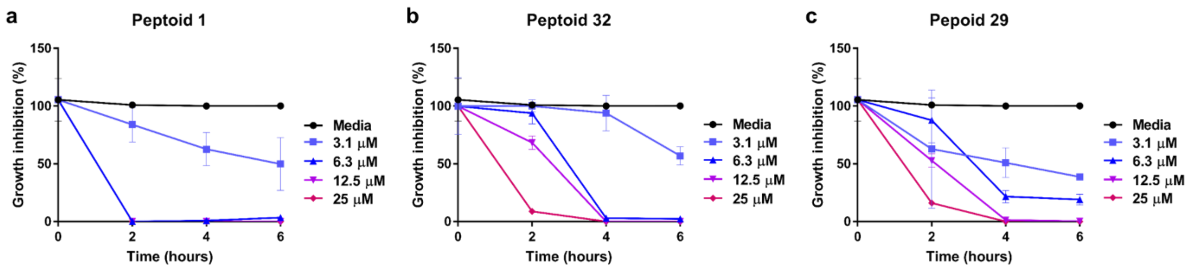

Figure S7. Effect of antimicrobial peptoids on bacterial growth kinetics. Growth inhibition of *P. aeruginosa* (KCTC 1637) are shown after incubation with peptoids: a) **1**, b) **32**, and c) **29**.

9. Real-time monitoring of *E. coli* treated with peptoid 29 using 3D optical diffraction tomography

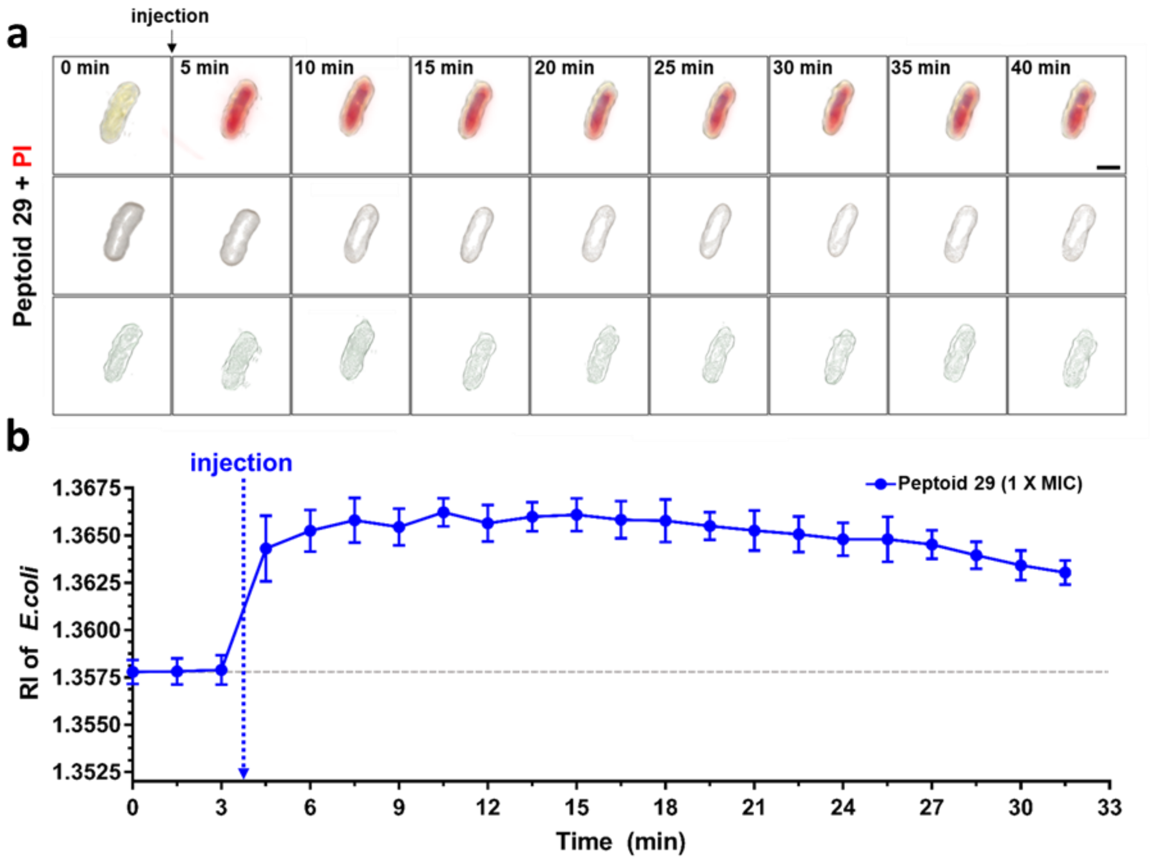

Figure S8. Real-time monitoring of *E. coli* treated with peptoid **29** (1 × MIC) using 3D optical diffraction tomography. a) Representative time-lapse 3D-rendered images of **29** (1 × MIC)-treated *E. coli* for 5 min interval during 40 min, overlaid with corresponding fluorescence images using propidium iodide (PI) (10 μg/mL). b) Quantitative analysis of time-lapse monitoring for mean RI values of *E. coli* treated with **29** (1 × MIC) for every 1.5 min interval during 30 min. Sample number: **29** (n = 8).

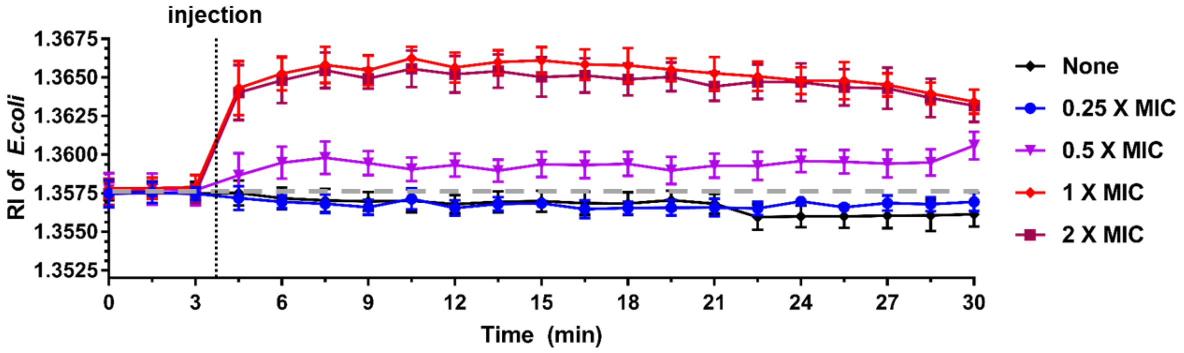

Figure S9. Quantitative analysis of time-lapse monitoring for mean RI values of non-treated and different concentrations of **29** (0.25, 0.5, 1, 2 × MIC)-treated *E. coli* at 1.5 min intervals over 30 min. Sample numbers were: None (n = 22), 0.25 × MIC (n = 9), 0.5 × MIC (n = 13) 1 × MIC (n = 8), and 2 × MIC (n = 10).

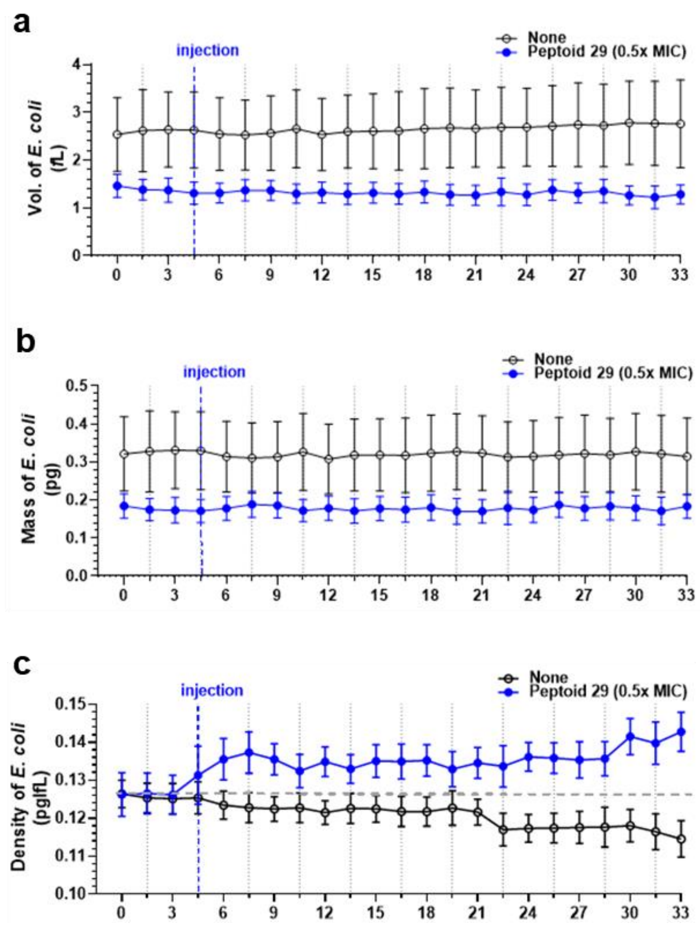

Figure S10. Quantitative analysis of time-lapse monitoring for a) volume, b) mass, and c) biomolecular density of control or **29** (0.5 × MIC)-treated *E. coli* at 1.5 min intervals over

30 min. Sample numbers were: control (n = 22) and **29** (n = 13). Measured RI values shown in Figure 3g and biomolecular density shown in (c) correlate well.

10. Representative fluorescent images and RI-based 3D rendered images of *E. coli* treated with melittin, buforin-II, and peptoid 1 in the presence of thioflavin

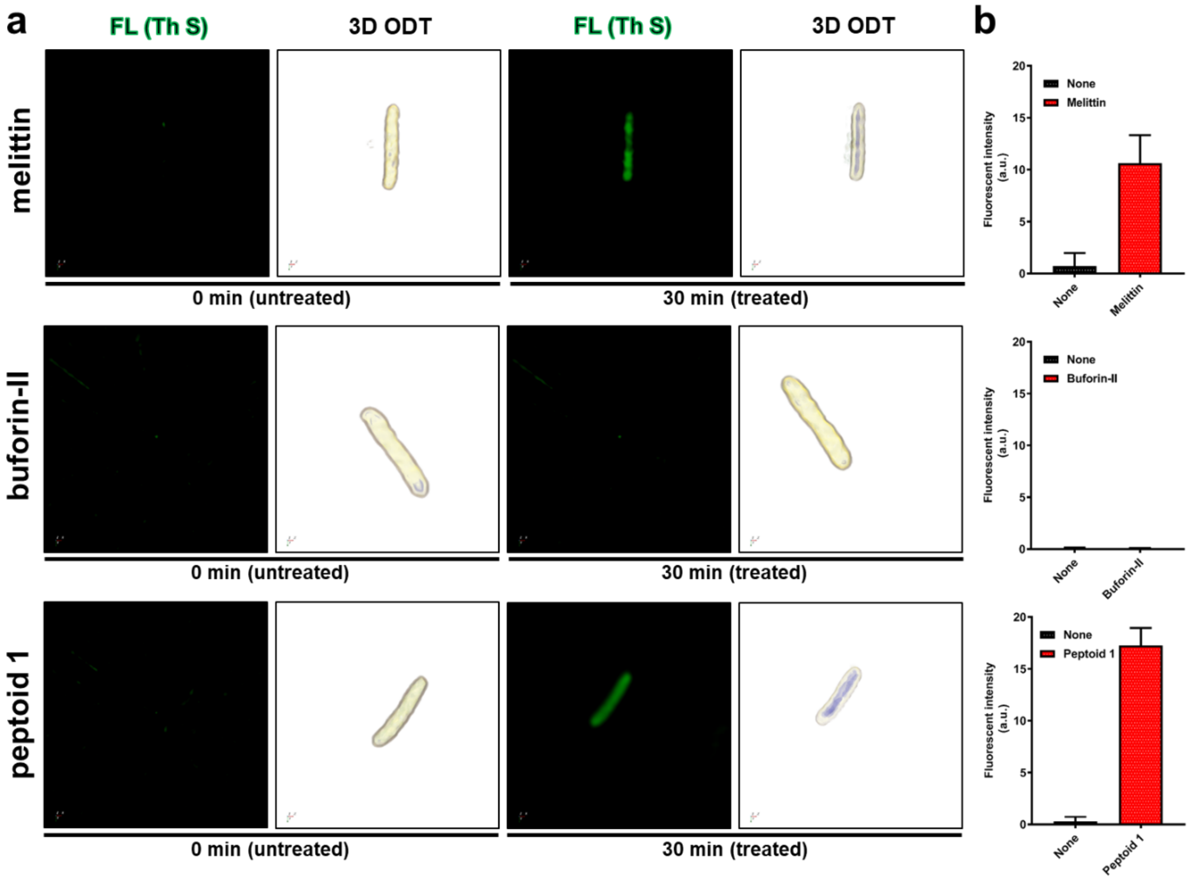

Figure S11. Effects of melittin, buforin-II, and peptoid **1** treatment on the morphology of *E. coli* (ATCC 25922) and on the thioflavin fluorescence. a) Fluorescence images using thioflavin S (Th S) and RI-based 3D rendered images of *E. coli*. Untreated (left) and treated cells (right). Control peptides (2 × MIC) and peptoid **1** (4 × MIC) were used. b) Quantitative analysis of Th S fluorescent intensity of *E. coli* treated with none, melittin, buforin-II, and peptoid **1**.

11. Bacterial respiration assay

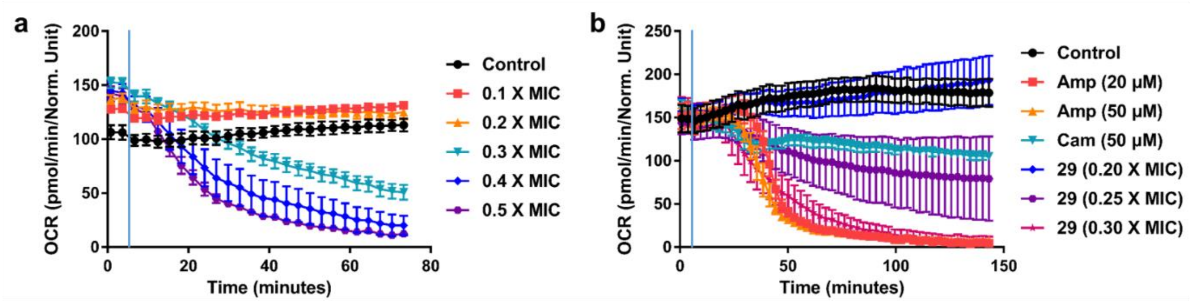

Figure S12. Real-time changes in oxygen consumption rate (OCR, in picomoles of molecular oxygen per minute) in response to treatment with peptoid **29**, ampicillin (Amp), and chloramphenicol (Cam) in *E. coli* measured on a Seahorse XFe 96 extracellular flux analyzer. The minimum inhibitory concentration (MIC) of **29** was 6.3 μM. a) Dose dependence of OCR changes in cells treated with **29** over time (2.5 min intervals). b) Comparison of OCR responses induced by **29**, Amp, and Cam treatment detected at 2.5 min intervals. The vertical line with azure color indicates the injection point of each antibiotic into the cultures. All data represent the mean ± SEM of 5-8 replicates.

Bacterial respiration assays

Cellular oxygen consumption rate (OCR) of *E. coli* ATCC 25922 was measured using a Seahorse XFe96 Extracellular Flux Analyzer (Agilent) as published previously.[9] Briefly, after overnight culture in LB, bacterial cells were diluted 1:200 into fresh M9 media (M6030, Sigma) and incubated for an additional 3 h at 37 °C. To conduct the measurements, bacteria were diluted to OD_{600} of 0.02 in M9 supplemented with 10 mM glucose. 90 μL of this cell suspension was loaded into a 96-well microplate precoated with poly-D-lysine (100 μg/mL, Sigma). Microplates were centrifuged for 10 min at 2,200 rpm to attach the cells, and 90 μL additional media was added to each well. To monitor uniform cell seeding, OCR baseline measurements were taken for two cycles before the automated injection of the indicated peptoid or antibiotics. OCR values were quantified every 2.5 min (mix and measure for 30 sec and 2 min, respectively) for an additional 48 cycles, and normalized to bacterial density measured at OD600.

12. Circular dichroism spectra

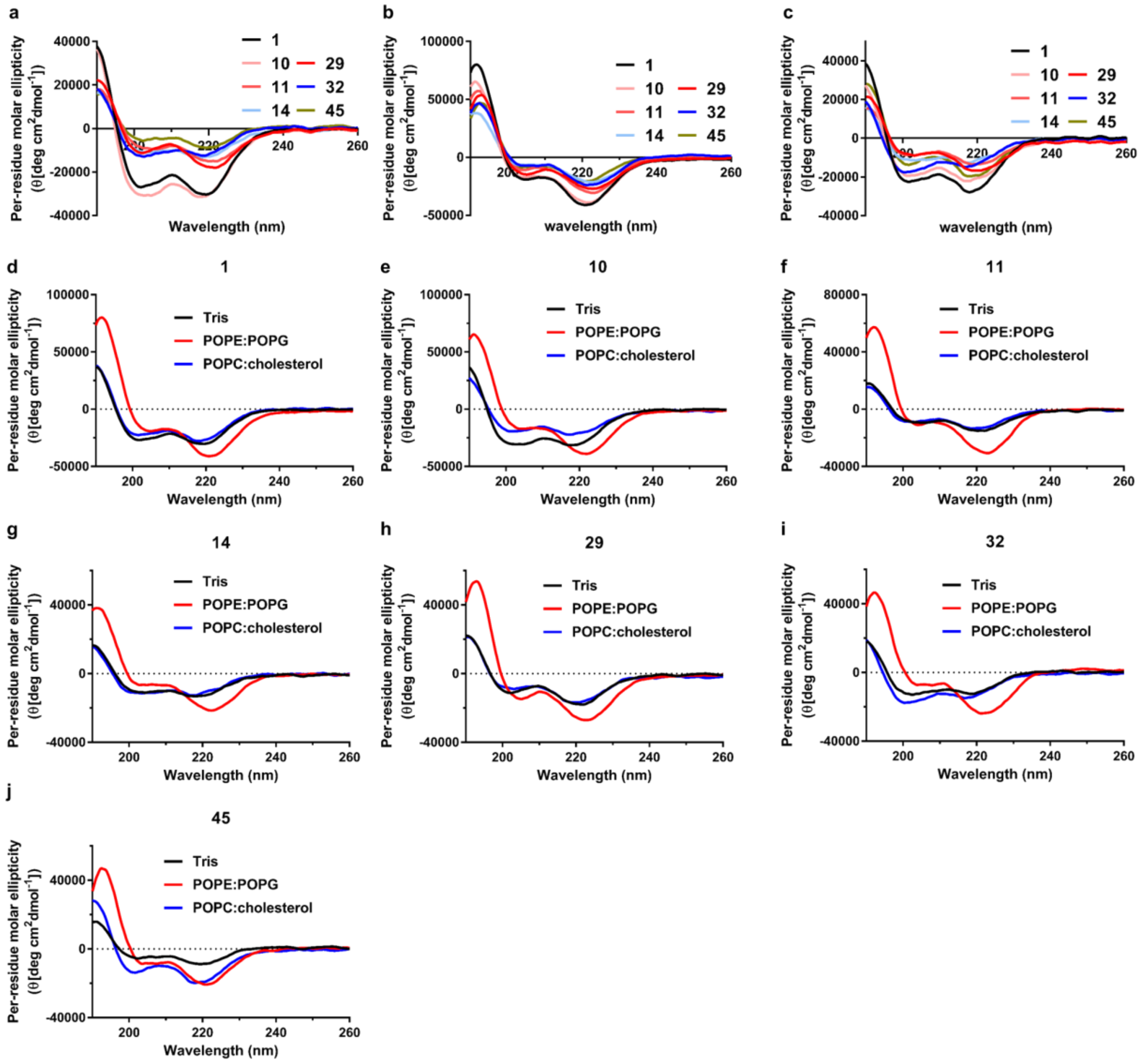

Figure S13. CD spectra of peptoids in the three environments: a) 5 mM Tris-HCl, b) 5 mM lipid vesicles (POPE/POPG=7:3 in molar ratio) suspended in 10 mM Tris-HCl, and c) 5 mM lipid vesicles (POPC/cholesterol=1:1 in molar ratio) suspended in 10 mM Tris-HCl. Conformational changes of peptoids d) **1**, e) **10**, f) **11**, g) **14**, h) **29**, i) **32**, and j) **45** shown in the three environments.

Circular dichroism (CD) spectroscopy for confirming the interaction between lipid vesicles and peptoids

CD measurements were performed on a Jasco model 815 spectropolarimeter using quartz cells with a path length of 0.2 mm. Scans were conducted with the following conditions: 100 nm/min speed, 260–190 nm, 0.2 nm data pitch, 1 nm bandwidth, 2 s response, and 100 mdeg sensitivity. The displayed data are the average of 40 successive spectral accumulations. Data were normalized in terms of per-residue molar ellipticity (deg·cm^2·dmol^{-1}). CD measurements were performed at a peptoid salt concentration of 50 μM in either 5 mM aqueous Tris-HCl buffer (pH 7.0) or 5 mM lipid vesicles in 10 mM aqueous Tris-HCl buffer (pH 7.0). All CD measurements were performed at 20 °C. Peptoid concentrations were determined using the dry weight of lyophilized material and were based on the TFA salt. To create samples for CD experiments in the presence of lipid vesicles, a solution of lipids in buffer (10 mM lipid, 20 mM Tris) was diluted to a final concentration of 5 mM lipids, 10 mM Tris by adding equal amounts of water and an aqueous peptoid stock solution.

Lipid vesicle preparation for circular dichroism (CD) experiments

Vesicles were prepared in small batches at 10 mM total lipid concentration in 20 mM aqueous Tris-HCl (pH 7.0) buffer. Vesicle suspensions were diluted to a final concentration of 5 mM lipids and 10 mM Tris buffer for CD experiments by adding water and aqueous peptoid stock solutions. Next, 1-palmitoyl-2-oleoyl-sn-glycero-3-phosphoethanolamine (POPE) and 1-palmitoyl-2-oleoyl-sn-glycero-3-phosphoglycerol (POPG) chloroform solutions (25 mg/mL) were combined at a 7:3 molar ratio in a round-bottom flask, and chloroform was removed using a rotary evaporator. The lipid film was hydrated with 20 mM Tris-HCl buffer at approximately 40 °C for one h with occasional vortexing. The lipid suspension was then sonicated at 40 °C using a 1.9 L Branson sonicator bath until the solution became clear (~30–45 min). The resulting vesicles were used in the CD experiments within 6 h of preparation. Vesicles of 1-palmitoyl-2-oleoylphosphatidylcholine (POPC):cholesterol were prepared at a 1:1 molar ratio using the same methods.

13. Small angle X-ray scattering (SAXS) data

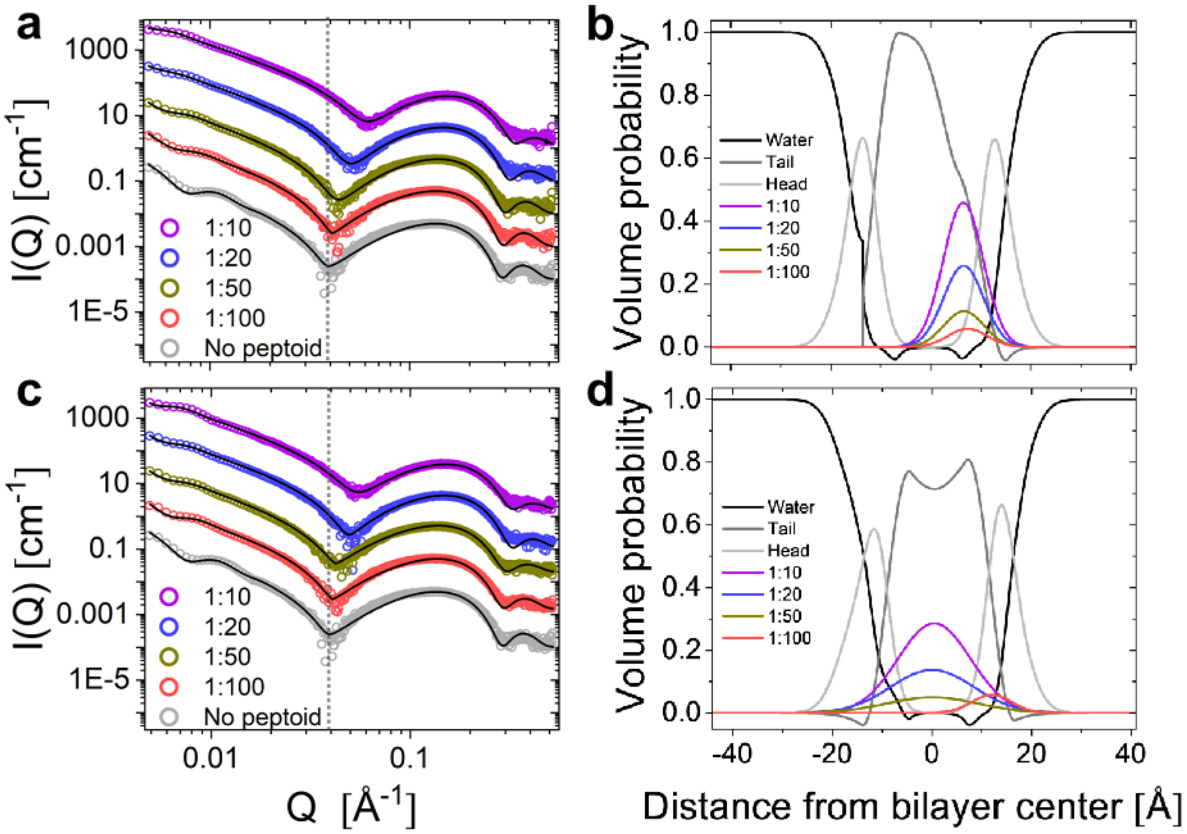

Figure S14. Membrane interaction of peptoids **11** and **29**. Small angle X-ray scattering (SAXS) data of peptoids mixed with lipid vesicles at different mixing ratio (peptoid:lipid molar ratios indicated in legends) plotted together with best model fit, and the resulting volume probability plot calculated from fit parameters. (a and b) **11** (c and d) **29**. A gray dotted line has been inserted in plot a) and c) to highlight the shift in the minima towards higher Q upon peptoid addition as compared to the neat liposomes. The SAXS curves in a) and d) have been offset with a factor of 10 for better visualization.

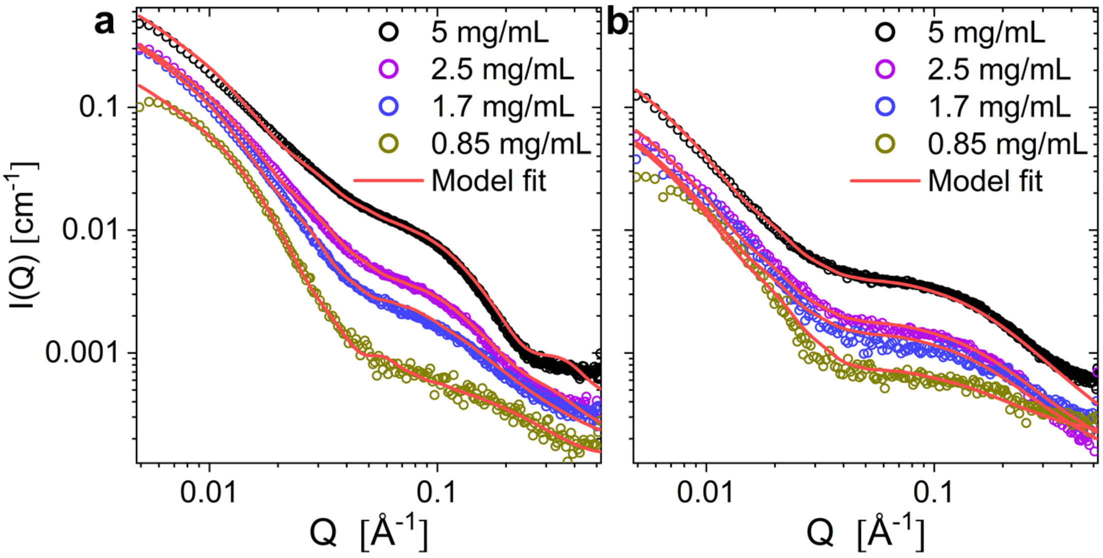

Figure S15. SAXS data revealing the concentration dependence of the self-assembled structure of peptoids. SAXS data on peptoids in aqueous environment at different peptoid concentrations (indicated in legends), plotted together with best fit. a) **11** b) **29**.

14. In vitro biological assays

Killing kinetics

The kinetics of antimicrobial activity against *E. coli* (ATCC 25922) were assessed at a peptoid concentration corresponding to 1×, 2× and 4 × MIC. Briefly, an overnight culture of bacteria was diluted at mid log-phase bacteria (2–5 × 10^5 cfu/mL) and incubated with peptoid with desired concentration in MHB2. The bacterial suspension was added to a 96-well polypropylene u- bottomed plate containing the peptoid. The plate was incubated without shaking at 37 °C for 4 h. Samples (20 µl) were taken at time 0.5, 1, 2 and 4 h and diluted in ice PBS buffer from which 100 µl was plated on LB agar plates. The plates were incubated for 18–24 hours at 37 °C and colony forming units (CFU) were counted. The experiment was conducted triplicates, and a curve was plotted between CFU and time (h).

NPN uptake assay

The ability of peptoids to increase the permeability of the outer membrane of Gram-negative bacteria was determined by measuring the incorporation of the NPN fluorescent dye into the outer membrane of *E. coli* ATCC 25922. Bacterial cells were suspended to a final concentration of OD_{600} = 0.05 in 5 mM HEPES buffer, pH 7.2, containing 5 mM KCN. Then

200 μL NPN dye was added to wells of a 96 well plate to produce a final concentration of 10 μM, and the background fluorescence was recorded (λ_{ex} = 350 nm, λ_{em} = 420 nm). Aliquots of peptoids were added to the well, and fluorescence was recorded as a function of time until there was no further increase in fluorescence. Increases of the permeability of the outer membrane after the addition of peptoids was reflected by increased fluorescence values as a result of NPN incorporation into the membrane.

ONPG hydrolysis assay

The permeability of the inner membrane was determined using *E. coli* ML-35 cells exhibiting *β*-galactosidase activity. The intact bacterial inner membrane is non-permeable to *o*-nitrophenyl-*β*-D-galactopyranoside (OPNG), a chromogenic substrate of the *β*-galactosidase. Following damage to the inner membrane, OPNG enters the cells and is metabolized by *β*-galactosidase creating a chromogenic substrate. *E. coli* cells were washed in 10 mM sodium phosphate (pH 7.4) containing 100 mM NaCl and resuspended in the same buffer at a final concentration of OD_{600} = 0.5 containing 1.5 mM ONPG. The hydrolysis of ONPG and generation of *o*-nitrophenol over time were monitored as absorbance change at 405 nm following the addition of peptoids.

S9 fraction assay

The resistance of peptoids against metabolic enzyme degradation was measured by an S9 fraction assay. Human liver S9 fraction is the 9000 g supernatant of a human liver homogenate containing mainly cytochromes P450 (CYPs 450), uridine 5'-diphosphoglucuronosyltransferase (UGTs), and various cytosolic enzymes. The peptoid was dissolved in autoclaved water, added to 100 mM pH 7.4 Tris-HCl buffer solution containing 3.3 mM magnesium chloride, S9 fractions, and nicotinamide adenine dinucleotide phosphate (NADPH) as a cofactor stimulating metabolism by CYPs 450. The prepared solution was incubated at 37 ^{o}C overnight. The desired amount of this solution was transferred into a quenching solution (MeCN + 0.1% TFA) at each specific time point. Quenched samples were centrifuged for 5 minutes at 1200 rpm and analyzed by analytical HPLC or LC-MS.

Protein extraction and quantification using Bradford assay

Bacteria were incubated overnight and sub-cultured when the optical density of the culture at λ = 600 nm reached at 0.5. The cultures were transferred to 50 mL conical tubes, and

bacteria were pelleted at 4,000 rpm for 10 minutes at 8 ^{o}C. The supernatant was removed and the pellet was suspended in 2 mL of PBS buffer. This suspension was transferred to a 15 mL conical tube and cooled on ice. The culture was sonicated for 5 sec and cooled again for 55 sec. This cycle was repeated 15 times. The lysate was centrifuged at 4,000 rpm for 20 minutes at 8 ^{o}C. The supernatant was transferred into a fresh 2 mL Eppendorf tube and stored at -80 ^{o}C. Protein quantification was performed using TaKaRa Bradford Protein Assay Kit according to the manufacturer's instructions.

DNA extraction and quantification

DNA extraction was performed using TaKaRa MiniBEST Bacteria Genomic DNA Extraction Kit Ver. 3.0 according to the manufacturer's instructions.

Protein and DNA aggregation assay

The flocculation of the proteins and DNA caused by peptoids was confirmed by the change in the fluorescent intensity of thioflavin T (Th T) that detects aggregated proteins and double stranded DNA. The appropriate combination of 500 μg/mL of protein solution, 50 ng/mL of genomic DNA, 25 μM of Th T, and 25 μM of peptoid **29** were added into a black 96-well plate in triplicate and incubated at 37 °C for an hour. After incubation, the fluorescence was measured using a microplate reader (excitation wavelength = 450 nm, emission wavelength = 485 nm).

15. References

[1] T. Uno, E. Beausoleil, R. A. Goldsmith, B. H. Levine, R. N. Zuckermann, *Tetrahedron Lett.* **1999**, *40*, 1475.

[2] A. P. Krapcho, C. S. Kuell, *Synth. Commun.* **1990**, *20*, 2559.

[3] R. N. Zuckermann, E. J. Martin, D. C. Spellmeyer, G. B. Stauber, K. R. Shoemaker, J. M. Kerr, G. M. Figliozzi, D. A. Goff, M. A. Siani, R. J. Simon, *J. Med. Chem.* **1994**, *37*, 2678.

[4] W. Huang, J. Seo, S. B. Willingham, A. M. Czyzewski, M. L. Gonzalgo, I. L. Weissman, A. E. Barron, *PLOS ONE* **2014**, *9*, e90397.

[5] a) A. M. Czyzewski, H. Jenssen, C. D. Fjell, M. Waldbrook, N. P. Chongsiriwatana, E. Yuen, R. E. Hancock, A. E. Barron, *PLoS One* **2016**, *11*, e0135961; b) J. A. Patch, A. E. Barron, *J. Am. Chem. Soc.* **2003**, *125*, 12092.
[6] J. Lee, D. Kang, J. Choi, W. Huang, M. Wadman, A. E. Barron, J. Seo, *Bioorg. Med. Chem. Lett.* **2018**, *28*, 170.
[7] K. Sikora, M. Jaśkiewicz, D. Neubauer, M. Bauer, S. Bartoszewska, W. Barańska-Rybak, W. Kamysz, *Amino Acids* **2018**, *50*, 609.
[8] A. Pini, L. Lozzi, A. Bernini, J. Brunetti, C. Falciani, S. Scali, S. Bindi, T. Di Maggio, G. M. Rossolini, N. Niccolai, L. Bracci, *Amino Acids* **2012**, *43*, 467.
[9] M. A. Lobritz, P. Belenky, C. B. Porter, A. Gutierrez, J. H. Yang, E. G. Schwarz, D. J. Dwyer, A. S. Khalil, J. J. Collins, *Proc. Natl. Acad. Sci. USA* **2015**, *112*, 8173.
